# Post-COVID symptoms are associated with endotypes reflecting poor inflammatory and hemostatic modulation

**DOI:** 10.3389/fimmu.2023.1243689

**Published:** 2023-08-23

**Authors:** Andy Yi An, Arjun Baghela, Peter G. Y. Zhang, Travis M. Blimkie, Jeff Gauthier, Daniel Elias Kaufmann, Erica Acton, Amy H. Y. Lee, Roger C. Levesque, Robert E. W. Hancock

**Affiliations:** ^1^ Centre for Microbial Diseases and Immunity Research, University of British Columbia, Vancouver, BC, Canada; ^2^ Département de microbiologie-infectiologie et d’immunologie, Université de Laval, Laval, QC, Canada; ^3^ Department of Medicine, Université de Montréal, Montréal, QC, Canada; ^4^ McGill Genome Centre, Fonds de recherche du Québec (FRQ) COVID-19 Biobank, Montreal, QC, Canada; ^5^ Department of Molecular Biology and Biochemistry, Simon Fraser University, Burnaby, BC, Canada

**Keywords:** long COVID, COVID-19, endotypes, gene expression, personalized medicine

## Abstract

**Introduction:**

Persistent symptoms after COVID-19 infection (“long COVID”) negatively affects almost half of COVID-19 survivors. Despite its prevalence, its pathophysiology is poorly understood, with multiple host systems likely affected. Here, we followed patients from hospital to discharge and used a systems-biology approach to identify mechanisms of long COVID.

**Methods:**

RNA-seq was performed on whole blood collected early in hospital and 4-12 weeks after discharge from 24 adult COVID-19 patients (10 reported post-COVID symptoms after discharge). Differential gene expression analysis, pathway enrichment, and machine learning methods were used to identify underlying mechanisms for post-COVID symptom development.

**Results:**

Compared to patients with post-COVID symptoms, patients without post-COVID symptoms had larger temporal gene expression changes associated with downregulation of inflammatory and coagulation genes over time. Patients could also be separated into three patient endotypes with differing mechanistic trajectories, which was validated in another published patient cohort. The “Resolved” endotype (lowest rate of post-COVID symptoms) had robust inflammatory and hemostatic responses in hospital that resolved after discharge. Conversely, the inflammatory/hemostatic responses of “Suppressive” and “Unresolved” endotypes (higher rates of patients with post-COVID symptoms) were persistently dampened and activated, respectively. These endotypes were accurately defined by specific blood gene expression signatures (6-7 genes) for potential clinical stratification.

**Discussion:**

This study allowed analysis of long COVID whole blood transcriptomics trajectories while accounting for the issue of patient heterogeneity. Two of the three identified and externally validated endotypes (“Unresolved” and “Suppressive”) were associated with higher rates of post-COVID symptoms and either persistently activated or suppressed inflammation and coagulation processes. Gene biomarkers in blood could potentially be used clinically to stratify patients into different endotypes, paving the way for personalized long COVID treatment.

## 1 Introduction

The COVID-19 pandemic has infected >650 million people as of June 2023 (1). While the death toll is alarmingly >6.5 million, what is just as alarming is the fact that a substantial proportion (12.7-43%) of survivors could develop persistent symptoms (2, 3) that decrease their quality of life, affect physical and cognitive function, and decrease their participation in society (4, 5). These symptoms include fatigue, shortness of breath, difficulty concentrating, loss of smell and taste, muscle pain, joint pain, and diarrhea, among almost 200 different symptoms (5). Various names have been given to this phenomenon, including “long COVID”, “chronic COVID”, “post-acute sequelae of SARS-CoV-2 infection”, and “post-COVID condition” (6). Both hospitalized and non-hospitalized patients are at risk of developing persistent symptoms (7), with hospitalized patients having slightly higher risk (2). This phenomenon does not seem to be unique to the SARS-CoV-2 virus, since influenza (8), Ebola (9), SARS-CoV-1 (10), and sepsis in general (“post-sepsis syndrome”) (11) also appear to be associated with persistent symptoms after discharge. However, a recent study suggested that seven sequelae (palpitations, hair loss, fatigue, chest pain, dyspnea, joint pain, and obesity) were more associated to COVID-19 than other common viral respiratory infections (12).

With such a large proportion of people infected worldwide, a significant number of people will be unable to return to work and will need to seek increased medical care, with severe long-term economic and healthcare implications (13). Long COVID is estimated to cost $16 trillion in just the United States, as a result of loss of productivity and increased healthcare access associated with premature death, long-term health impairment, and mental health impairment (14). Thus, it is imperative to understand how and why patients develop these symptoms.

Despite its prevalence, the pathophysiology of long COVID is still not well understood, and the non-specific nature of its clinical manifestations makes targeted investigations of potential mechanisms challenging, although multiple mechanisms have been proposed. Permanent inflammatory damage to multiple organ systems during the acute disease period has been proposed to be one potential cause, particularly for neurologic and respiratory symptoms (7). Chronic inflammation could also be detrimental, with inflammatory cytokines documented to be elevated for months after infection in patients with post-COVID symptoms (15, 16). Anti-phospholipid autoantibodies can potentially lead to later cardiovascular complications (17), while anti-interferon antibodies (18) and anti-nuclear antibodies (19) have been associated with post-COVID symptoms. Failure to fully clear the SARS-CoV-2 virus (20, 21) or reactivation of latent viruses (22) may result in chronic infections. Lastly, abnormal coagulation mechanisms, resulting in “microclots”, have been attributed to the development of long-term symptoms (23).

Few analyses of whole blood gene expression have been performed to compare gene expression trajectories of patients with or without persistent post-COVID symptoms. A cohort of 69 patients (24) demonstrated evidence of transcriptomic dysregulation up to 24 weeks post discharge in patients with persistent symptoms; however, this study only profiled patients after discharge and did not have data on these patients while hospitalized. A cohort of 165 patients assessed in-hospital differences between patients with or without symptoms 1 year after discharge, which found a relationship between specific symptoms, immunoglobulin-related genes, and plasma cells in hospital, but did not provide gene expression data at follow-up for comparison (25).

In this study, we performed whole blood RNA-Seq on samples collected from COVID-19 survivors both in hospital and after discharge to identify gene expression changes over time between patients with and without post-COVID symptoms. Patients without post-COVID symptoms demonstrated resolution of immune and hemostatic pathways from hospitalization to follow-up. We were also able to classify patients into three endotypes, which we named “Resolved”, “Suppressive”, and “Unresolved”, reflecting the trajectories of immune and hemostatic processes from hospital to follow-up. The “Suppressive” and “Unresolved” endotypes were associated with a higher proportion of post-COVID symptoms, highlighting that the mounting and subsequent resolution of immune and hemostatic responses were key to preventing symptoms after discharge. Whole blood gene biomarkers for long COVID endotypes were also identified, which could potentially be used to guide personalized treatment and prognosis.

## 2 Methods

### 2.1 Sample collection

Through the Banque Québécoise de la COVID-19 (BQC19) biobank (26), 24 adult (36-84 years old, median age 59 years, 17/24 male) patients who were hospitalized in Quebec, Canada, primarily due to pulmonary disease from SARS-CoV-2 infection (*e.g.*, COVID-19 pneumonia) were enrolled in this study. Sample size estimation and power analysis was performed using the package *ssizeRNA* (v1.3.2) (27) to show that this sample size was sufficiently powered (power = 0.8, false discovery rate = 0.05) to detect differentially expressed (DE) genes (**Figure S1C**). Approximately 2.5mL of whole blood from each patient was collected into PAXgene Blood RNA tubes (BD Biosciences) at two time points: <10 days post-hospital admission, and at a follow-up visit (4-12 weeks post-hospital discharge) (**Figure S1A**). Patients self-reported any persistent symptoms related to COVID-19 at follow-up. Ten patients reported at least one persistent symptom that developed after COVID-19 (**Figure S1B**). All samples were collected between July 2020 and May 2021, suggesting these patients likely were infected with the ancestral strain, or either the Alpha or Beta variants. No patients were vaccinated prior to hospital admission. RNA was extracted from whole blood and RNA-Seq was performed as described previously (28): total RNA was extracted with the PAXgene Blood RNA Kit (Qiagen), poly-adenylated RNA was enriched using NEBNext Poly(A) mRNA Magnetic Isolation Module (NEB), and cDNA libraries were prepared using the NEBNext RNA First Strand Synthesis Module, NEBNext Ultra Directional RNA Second Strand Synthesis Module, and NEBNext Ultra II DNA Library Prep Kit for Illumina (NEB). RNA-Seq was then performed at a depth of 50M reads/sample on an Illumina NovaSeq 6000 S4 instrument of 100 base-pair long paired-end sequence reads (excluding adapter/index sequences). Raw gene expression data can be found in GSE221234 and GSE222253.

### 2.2 Bioinformatics analysis and statistics

The sequencing data processing protocol included quality control using *FastQC* (v0.11.9) (29) and *MultiQC* (v1.6) (30), alignment to the human genome (Ensembl GRCh38.104) using *STAR* (v2.7.9a) (31), and read count assessments using *HTSeq* (v0.11.3) (32). All downstream bioinformatics analyses were done in R (v4.2.2). Hemoglobin associated genes and low read count genes (mean count <10) were filtered out, resulting in a gene universe of 18,826 Ensemble IDs for analysis. The package *DESeq2* (v1.34.0) was used to identify differentially expressed (DE) genes between patients with and without persistent post-COVID symptoms in hospital and at follow-up (Wald statistics model) (33). DE genes were defined as genes with an absolute fold change ≥1.5 and an adjusted-p-value <0.05 (Benjamini-Hochberg multiple test correction). The package *variancePartition* (v1.28.3) (34) was used to determine potential confounders to include in the DESeq2 model (**Figure S1G**): age, sex, sequencing batch, days in hospital, and days from discharge to follow-up sampling time (follow-up samples) or from hospital admission to in-hospital sampling time (in-hospital samples). A pair-wise analysis between hospital and follow-up samples of each patient was performed to identify gene expression trajectories; this was performed by investigating the effect of time in the patients with and without post-COVID symptoms, with individuals nested (as outlined in the DESeq2 vignette) (33). Essentially, patients were indexed to their previous sample, which controlled for individual underlying baseline differences (*e.g.*, genetics, comorbidities, etc.).

Pathway enrichment on up- and down-regulated DE genes was subsequently performed. The Reactome database is an open-source, peer-reviewed pathway database (35). To enable enrichment of more specific and biologically relevant Reactome pathways, DE genes were analyzed using the *SIGORA* package (v3.1.1) (36), which decreases the chance of observing multiple similar and overlapping pathways by analyzing gene pairs rather than individual genes (which may be present in overlapping pathways). Reactome pathways were considered significantly enriched with an adjusted p-value <0.001 (Bonferroni multiple test correction) as was recommended in *SIGORA*. These analyses were further supplemented by enrichment of Hallmark gene sets (gene sets that represent “specific, well-defined biological states or processes with coherent expression” from the Molecular Signatures database) (37) using *clusterProfiler* (v4.2.2) (38), with significant gene sets having an adjusted-p-value <0.05 (Benjamini-Hochberg multiple test correction).

To identify endotypes in follow-up patients, K-medoids unsupervised clustering using the package *cluster* (v2.1.4) (39) was performed using variance-stabilized-transformed counts, scaled across all samples for each gene. The process of K-medoids clustering, using the “Partitioning Around Medoids” algorithm, is as follows. First, *k* representative central samples (medoids) are selected, then the total Manhattan distance of the resulting clustering around the medoids is assessed and compared to distances of clustering using other medoids. This is repeated until the medoids that minimize the total clustering distances are ultimately selected (40). K-medoids was chosen over a similar clustering approach, K-means, due to its non-sensitivity to outliers and reduction of noise (40), and sepsis endotypes from our previous work were also identified through K-medoids clustering (41). Clustering metrics using K-medoids clustering showed that the optimal cluster number was *k* = 3 based off total within sum of square and the gap statistic. DE analysis was performed comparing these clusters/endotypes to each other at follow-up, in hospital, and over time, and DE genes were used for pathway enrichment as described above.

Gene signatures for these endotypes were identified by feature selection using LASSO regression from the package *glmnet* (v4.1.6) (42). These gene signatures and Hallmark gene sets were assessed using gene set variation analysis (GSVA) using the package *GSVA* (v1.46.0) (43). GSVA is a non-parametric, unsupervised method that calculates enrichment scores of gene sets (*e.g.*, pathways or signatures), allowing direct comparison of gene set enrichment in different groups (43). *CIBERSORTx*, a cell deconvolution method, was used to estimate cell proportions of 22 different cell types based on gene expression data with the LM22 marker set (44). Wilcox tests were performed when comparing GSVA scores and estimated cellular proportions as the data was non-parametric.

## 3 Results

### 3.1 Persistent Post-COVID symptoms were associated with worse quality of life

Ten of the 24 patients had persistent post-COVID symptoms >4 weeks post-discharge (termed “symptomatic”), and the most common symptoms in these symptomatic patients were fatigue and dyspnea (**Figure S1B**). The presence of these symptoms at follow-up was associated with lower quality of life, with patients reporting more difficulty with mobility (especially climbing stairs), having more pain and discomfort, feeling more breathless, and overall being frailer (**Table 1**). Notably, these patients did not statistically differ in various metrics of clinical severity in hospital [*e.g.*, highest recorded SOFA score and World Health Organization COVID-19 Clinical Progression Score (45)], cell proportions, lab values, treatments received, rates of ICU admission, and hospitalization duration compared to patients without post-COVID symptoms (termed “asymptomatic”) (**Table 1**), which was consistent with the literature indicating that the presence of these post-COVID symptoms is not associated with disease severity (46), since many mild and even non-hospitalized patients can also develop persistent symptoms (7, 8). In addition, common confounders including age and sex, as well as the time between discharge date and follow-up date, were not statistically different between these two groups. Interestingly, while diabetes and hypertension have been shown to be associated with poor outcomes during COVID-19 hospitalization (47), in this cohort, a greater proportion of asymptomatic patients had pre-existing diabetes and/or hypertension compared to symptomatic patients (**Table 1**). There is conflicting data on whether diabetes is a risk factor for developing post-COVID symptoms (48, 49). Thus, in this cohort, there did not appear to be clear clinical risk factors predisposing patients to develop post-COVID-19 symptoms, warranting further investigation into potential gene expression biomarkers.

**Table 1:** Demographics of patients with and without post-COVID-19 symptoms.

Clinical Variables	No Post-COVID-19 Symptoms (14)	Post-COVID-19 Symptoms (10)	P-value
Age	60.1 ± 12.2 (14)	54.5 ± 12.5 (10)	0.306
Sex (Male)	85.7% (12/14)	50.0% (5/10)	0.085
Body Mass Index	30.6 ± 6.5 (12)	28.7 ± 6.9 (7)	0.526
Admitted to ICU (Yes)	28.6% (4/14)	30.0% (3/10)	1.000
Smoker (Yes)	25.0% (3/12)	0.0% (0/7)	0.263
Comorbidities			
Asthma (Yes)	14.3% (2/14)	10.0% (1/10)	1.000
COPD (Yes)	0.0% (0/14)	10.0% (1/10)	0.417
Chronic Lung Disease (Yes)	21.4% (3/14)	10.0% (1/10)	0.615
Hypertension (Yes)	64.3% (9/14)	10.0% (1/10)	**0.013**
Diabetes (Yes)	50.0% (7/14)	0.0% (0/10)	**0.019**
Immunosuppressed (Yes)	21.4% (3/14)	10.0% (1/10)	0.615
Worst Laboratory Values			
Highest %Neutrophil	80.3 ± 7.9 (14)	77.0 ± 8.0 (10)	0.279
Lowest %Lymphocyte	6.8 ± 3.7 (14)	6.8 ± 2.9 (10)	0.838
Highest %Monocyte	8.6 ± 2.6 (14)	9.2 ± 1.7 (10)	0.364
Lowest Platelets (10^3^/µL)	244.4 ± 120.9 (14)	228.4 ± 100.3 (10)	0.884
Highest Estimated SOFA Score	3.3 ± 2.6 (14)	4.1 ± 3.1 (10)	0.456
Highest WHO COVID-19 Score	5.6 ± 1.4 (14)	6 ± 1.4 (10)	0.360
Hospitalized Duration (Days)	16.4 ± 14.8 (14)	14.9 ± 14.5 (10)	0.907
Follow-up Metrics			
Discharge to Follow-up (Days)	53.6 ± 13.7 (14)	45.3 ± 15 (10)	0.135
Mobility Score	0.1 ± 0.3 (14)	0.9 ± 0.9 (10)	**0.005**
Self-Care Score	0 ± 0 (14)	0.2 ± 0.6 (10)	0.272
Usual Activity Score	0.1 ± 0.3 (14)	0.4 ± 0.8 (10)	0.333
Pain and Discomfort Score	0.2 ± 0.4 (14)	1.1 ± 1 (10)	**0.012**
Anxiety and Depression Score	0.1 ± 0.4 (14)	0.4 ± 0.7 (10)	0.341
Breathlessness Score	0.4 ± 0.5 (14)	1.6 ± 0.8 (10)	**0.001**
Difficulty Carrying 10 Pounds	0.1 ± 0.4 (14)	0.7 ± 0.9 (10)	0.113
Difficulty Walking Across Room	0 ± 0 (14)	0.2 ± 0.4 (10)	0.099
Difficulty Climbing 10 Stairs	0 ± 0 (14)	0.7 ± 0.8 (10)	**0.004**
Difficulty Transferring from Chair to Bed	0.1 ± 0.3 (14)	0.3 ± 0.7 (10)	0.359
Number of Falls in Past Year	0.1 ± 0.4 (14)	0.1 ± 0.3 (10)	0.798
Total Frailty Score	1.2 ± 1.4 (14)	6.6 ± 5.8 (10)	**0.002**
Poor Health Self-Rating	15.7 ± 10.2 (14)	26.6 ± 13.3 (10)	0.051

For categorical variables, significance was tested using the Chi-squared test with Yates’s correction, or the Fisher’s exact test if any expected value was <5, and the percentage and fraction of patients fitting the category is displayed. For continuous variables, the Wilcoxon Rank-Sum test was used, and the mean ± standard deviation of the variable is displayed, with the number of patients assessed in brackets. Follow-up metric scores are discussed in **Table S1**. The full set of assessed clinical variables, including non-significant differences in arrival values, other comorbidities, and treatments administered in hospital, are found in **Table S2**. Significant p-values (p <0.05) are bolded.

### 3.2 Follow-up and in-hospital samples were transcriptionally indistinguishable between patients with and without post-COVID symptoms

To determine underlying pathophysiological differences between patients with and without persistent post-COVID symptoms, differential expression analysis was performed on the follow-up samples collected after discharge using the package *DESeq2 (* 33). Only two differentially expressed (DE) genes were identified when comparing patients who reported post-COVID symptoms and those who did not, which were *GYPE* (glycophorin E, part of the MNS blood group) and *ALDH1A1* (aldehyde dehydrogenase 1 family member A1) (**Figure S1D**), even after correcting for potential confounders (**Figure S1G**) and despite the comparison being adequately powered for DE gene detection (**Figure S1C**).

In addition, no DE genes were identified between in-hospital samples of patients who developed or did not develop symptoms after discharge (**Figure S1E**). Thus, whether a patient will develop post-COVID symptoms did not appear to be discernible based on responses while a patient was still in hospital. In contrast, comparisons based on disease severity (*e.g.*, whether a patient was admitted to the ICU or not) yielded many more DE genes (444 genes) (**Figure S1F**), consistent with studies linking disease severity to gene expression in hospitalized patients (41).

Overall, the lack of DE genes in comparisons using either follow-up or in-hospital samples indicated that either the transcriptomic signature associated with the presence or absence of post-COVID symptoms was not substantial, or that gene expression differences might have been masked by heterogeneity within individual patients, due to factors that might include inherent genetic differences, comorbidities, microbiome, diets, and treatments administered. The high residuals when looking at sources of variance in gene expression (using *variancePartition*) further supported this idea of uncaptured individual heterogeneity (**Figure S1G**). Thus, post-COVID effects on gene expression could not be fully captured by directly comparing symptomatic and asymptomatic patients. This might also result in part from different underlying pathophysiology that might reflect endotypes. To examine the first possibility of heterogeneity, these individual factors were taken into account by performing a trajectory analysis in which each patient was indexed to a previous sample from themselves.

### 3.3 Lack of post-COVID symptoms was associated with potential resolution of immune and hemostasis dysregulation over time

**Figure 1:**
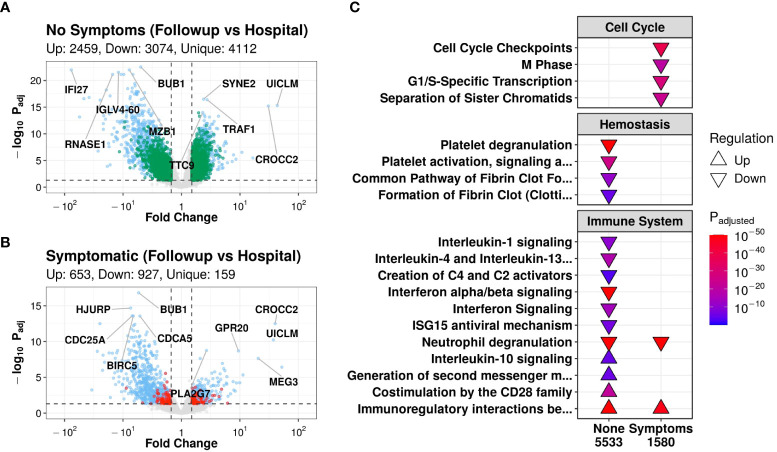
Patients without symptoms at follow-up had transcriptomic evidence of temporal immune and hemostatic resolution. Volcano plots of differentially expressed (DE) genes over time in patients without **(A)** and with **(B)** post-COVID symptoms. Coloured dots indicate DE genes, with green and red dots indicating DE genes that were uniquely DE in asymptomatic vs. symptomatic patients, respectively (blue dots indicate shared DE genes). The top 5 up- and down- regulated genes (lowest adjusted p-value and highest fold change) are labelled. **(C)**: Subset of notable enriched Reactome pathways from DE genes over time in patients who had post-COVID symptoms (“Symptoms”) or who did not have symptoms (“None”) at discharge, with all enriched pathways shown in **Figure S2A**. Pathways were considered “upregulated” (Δ) if the genes in this pathway were overrepresented in upregulated DE genes when compared to their prevalence in the genome, and vice versa for “downregulated” (∇). P-values were adjusted for multiple comparisons using Bonferroni, with an adjusted p-value cutoff <0.001. The total number of DE genes in each comparison are shown under each label.

All 24 patients had both an in-hospital and a follow-up sample, allowing the analysis of gene expression trajectories (*i.e.*, gene expression changes over time from hospital to discharge). In contrast to the single time point comparisons with few to no DE genes (**Figures S1D, E**), patients who were asymptomatic at follow-up had 5,533 DE genes over time, of which 4,112 genes were DE over time only in this group (**Figure 1A**). On the other hand, symptomatic patients had substantially less DE genes over time (1,580), of which only 159 were unique to symptomatic patients (**Figure 1B**).

Pathway enrichment of DE gene trajectories in asymptomatic patients showed enrichment of pathways from the Reactome database (35) involved in the immune system, hemostasis, and signal transduction (**Figure 1C**). Notably, down-regulated genes were enriched in hemostasis pathways such as “Platelet degranulation”, “Platelet activation, signaling, and aggregation”, “Common pathway of fibrin clot formation”, and “Formation of fibrin clot”; interleukin pathways such as “Interleukin-1 signaling” and “Interleukin-4/13 signaling”; the complement pathway “Creation of C4 and C2 activators”; and antiviral pathways such as “Interferon signaling”, “Interferon α/β signaling”, and “ISG15 antiviral mechanism” (**Figure 1C**). The down-regulation of these immune pathways suggested a decrease in the activity of multiple inflammatory processes, which was further supported by the upregulation over time of the anti-inflammatory pathway “Interleukin-10 signaling” (**Figure 1C**). In particular, these hemostasis and inflammatory pathways have been shown in the literature to be largely upregulated in hospitalised COVID-19 patients (50–52). Thus, the observed down-regulation over time may suggest a return to homeostasis after discharge in only patients who did not have post-COVID symptoms at follow-up. Conversely, adaptive immune pathways, such as the T cell signaling pathways “Generation of second messenger molecules” and “Co-stimulation by the CD28 family”, increased over time in asymptomatic patients (**Figure 1C**). Considering that adaptive responses have been shown in the literature to be suppressed in hospitalized COVID-19 patients (53), upregulation over time again suggested a return to immune homeostasis. Both groups, however, demonstrated down-regulation over time of the “Neutrophil degranulation” pathway (which can be related to inflammation) and upregulation of the adaptive pathway “Immunoregulatory interactions between a lymphoid and a non-lymphoid cell”, suggesting that even symptomatic patients potentially had some level of immune resolution as they recovered, at least for these two pathways (**Figure 1C**).

In symptomatic patients, a large proportion of enriched pathways that were altered from hospitalization to follow-up related to “Cell Cycle” pathways, which were enriched in down-regulated genes (**Figure 1C**). These changes in cell cycle pathways prompted further investigation into estimated cell proportions using *CIBERSORTx*, a computational cell deconvolution technique using gene expression data (44). Interestingly, only asymptomatic patients had a significant decrease in neutrophil proportions over time (**Figure S2C**), consistent with the overall down-regulation of inflammatory pathways (**Figure 1C**).

To validate the Reactome pathway results, enrichment was also performed using Hallmark gene sets (37). In asymptomatic patients, genes that were downregulated over time were enriched for the “Inflammatory response”, “Interferon-α response”, “Interferon-γ response”, “IL6-JAK-STAT3 signaling”, “Complement”, “TNFα-signaling via NF-kB”, and “Coagulation” gene sets, while upregulated genes were enriched for adaptive gene sets such as “IL2-STAT5 signaling” and “Allograft rejection” (**Figure S2B**), consistent with the above-described immune- and hemostasis-related Reactome pathway enrichment results.

Based on these trajectory analyses, it appeared that large temporal gene expression changes were associated with a lack of post-COVID symptom development. These changes reflected a decrease in activity of inflammatory and hemostasis pathways and an increase in the activity of adaptive immune pathways, which have been documented to be respectively elevated (50) and suppressed (53) during severe COVID-19. Thus, these results are consistent with asymptomatic patients returning to homeostasis with respect to immune and hemostatic function. Conversely, fewer DE genes over time were found in patients with persistent symptoms, consistent with a reduced return to homeostasis. This could indicate either a failure of these processes to resolve, or further heterogeneity within this group of patients that confounded this comparison.

### 3.4 Three mechanistically distinct endotypes were identified in follow-up patients

A second possible source of variation explaining the lack of DE genes in direct comparisons of patients with or without post-COVID symptoms, as well as fewer DE genes over time in symptomatic patients, could be the presence of endotypes. Endotypes are groups of patients with distinct pathophysiological mechanisms. Previous work from our lab employed the use of K-medoids clustering, an unsupervised machine-learning clustering algorithm, to identify endotypes in early sepsis (41). Here, we used this approach to cluster patients at follow-up into endotypes. Based on optimal clustering metrics (gap statistic and total within sum of square), the optimal number of clusters was determined to be three (**Figures S3A, B**). These clusters/endotypes were named “Resolved”, “Suppressive”, and “Unresolved” based on the trajectories of inflammatory and hemostatic processes that are described below. Furthermore, these endotypes were then validated in an independent cohort of 65 patients (GSE169687) (24) as described below.

**Figure 2:**
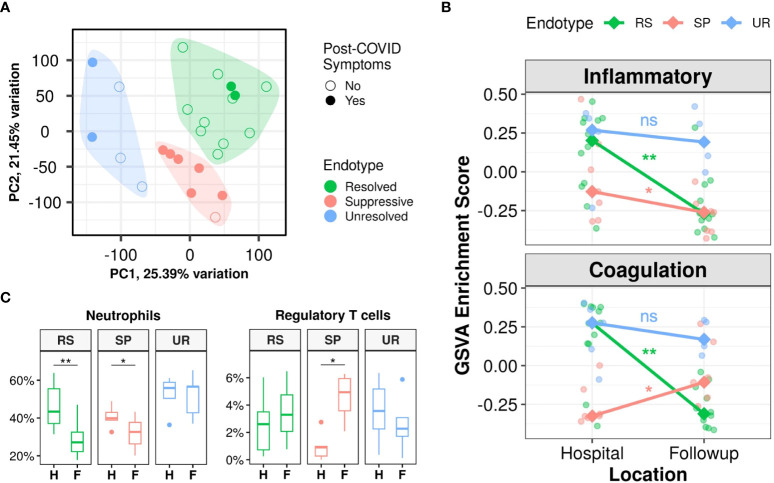
Three post-discharge endotypes (Resolved, Suppressive, and Unresolved) showed distinct immune and hemostasis trajectories and differing rates of post-COVID symptoms. **(A)**: Principal component analysis indicating separation of follow-up samples into three clusters/endotypes based on K-medoids clustering, highlighted by the coloured polygons, with most patients with post-COVID symptoms falling in the Suppressive endotype (red), while the Resolved endotype (green) was mostly composed of patients without symptoms after discharge. **(B)**: Differing trajectories of the Resolved, Suppressive, and Unresolved endotypes based on gene set variation analysis (GSVA) enrichment scores of the Hallmark “Inflammatory Response” and “Coagulation” gene sets. Trend lines connect the median enrichment scores of each endotype from hospital to follow-up, points indicate individual sample enrichment scores. **(C)**: Estimated proportions of neutrophils and regulatory T cells using *CIBERSORTx*, with all cell types in **Figure S4C**. Pair-wise Wilcoxon rank-sum test between in-hospital and follow-up samples for GSVA enrichment scores and cell proportions were performed to determine significance and adjusted for multiple corrections (Benjamini-Hochberg): **p<0.01, and *p<0.05, ns = not significant. H, Hospital samples; F, Follow-up samples; RS, Resolved; SP, Suppressive; UR, Unresolved.

Interestingly, the proportion of patients with post-COVID symptoms differed significantly (p=0.015) between the three endotypes. Almost all the patients in the Suppressive endotype had post-COVID symptoms (85.7%) and a substantial proportion was also seen in the Unresolved endotype (40%), while the lowest proportion was seen in the Resolved endotype (16.7%) (**Figure 2A**; **Table 2**). Other than the presence of post-COVID symptoms, metadata variables that were significantly different across the three endotypes were body mass index (lowest in Unresolved, p=0.01), highest recorded creatinine (reflecting kidney function) during hospitalization (highest in Unresolved, p=0.034), and corticosteroid use during hospitalization (lowest rate in Resolved, p=0.038) (**Table 2**). Notably, age, sex, severity of active disease (based on hospitalization duration, ICU admission, highest recorded SOFA/WHO score), and time between discharge and follow-up sampling were not significantly different between endotypes.

**Table 2:** Patient demographics of patients in the three endotypes.

Clinical Variables	Resolved (12)	Suppressive (7)	Unresolved (5)	P-value
Age	60.5 ± 13.7 (12)	53.7 ± 13.6 (7)	56.8 ± 6.1 (5)	0.535
Sex (Male)	75.0% (9/12)	71.4% (5/7)	60.0% (3/5)	0.850
Body Mass Index	33.2 ± 6.9 (10)	29.3 ± 3.2 (4)	23.9 ± 2 (5)	**0.010**
Admitted to ICU (Yes)	33.3% (4/12)	28.6% (2/7)	20.0% (1/5)	1.000
Hospitalized Duration (Days)	18.2 ± 14.8 (12)	15 ± 17.9 (7)	10.8 ± 7.6 (5)	0.381
Post-COVID Symptoms (Yes)	16.7% (2/12)	85.7% (6/7)	40.0% (2/5)	**0.015**
Discharge to Follow-up (Days)	54.1 ± 14.2 (12)	45.7 ± 10 (7)	46.8 ± 20.4 (5)	0.395
Smoker (Yes)	20.0% (2/10)	0.0% (0/5)	25.0% (1/4)	0.561
Worst Laboratory Values				
Highest %Neutrophil	0.8 ± 0.1 (12)	0.7 ± 0.1 (7)	0.8 ± 0.1 (5)	0.091
Lowest %Lymphocyte	0.1 ± 0 (12)	0.1 ± 0 (7)	0.1 ± 0 (5)	0.217
Highest %Monocyte	0.1 ± 0 (12)	0.1 ± 0 (7)	0.1 ± 0 (5)	0.276
Lowest Platelets (10^3^/µL)	253 ± 122 (12)	183 ± 61 (7)	278 ± 128 (5)	0.245
Highest Creatinine	85.1 ± 42.3 (12)	63.9 ± 9.8 (7)	171± 192 (5)	**0.034**
Highest Estimated SOFA Score	3.7 ± 2.8 (12)	2.9 ± 2.9 (7)	4.6 ± 3.1 (5)	0.273
Highest WHO COVID-19 Score	5.8 ± 1.4 (12)	5.6 ± 1.6 (7)	6.0 ± 1.2 (5)	0.617
Treatments During Hospitalization				
Antifungal (Yes)	8.3% (1/12)	14.3% (1/7)	0.0% (0/5)	1.000
Antibiotics (Yes)	83.3% (10/12)	71.4% (5/7)	80.0% (4/5)	0.819
Antiviral				
Lopinavir/Ritonavir (Yes)	33.3% (4/12)	0.0% (0/7)	0.0% (0/5)	0.144
Remdesivir (Yes)	0.0% (0/12)	28.6% (2/7)	0.0% (0/5)	0.112
Other Antiviral (Yes)	8.3% (1/12)	0.0% (0/7)	20.0% (1/5)	0.457
Immunomodulator				
Systemic Corticosteroids (Yes)	41.7% (5/12)	85.7% (6/7)	100.0% (5/5)	**0.038**
Tocilizumab (Yes)	0.0% (0/12)	14.3% (1/7)	40.0% (2/5)	0.057
Sarilumab (Yes)	8.3% (1/12)	0.0% (0/7)	0.0% (0/5)	1.000
Other Immunomodulator (Yes)	16.7% (2/12)	0.0% (0/7)	20.0% (1/5)	0.564
Other Treatments				
Vasopressor support (Yes)	8.3% (1/12)	14.3% (1/7)	0.0% (0/5)	1.000
Prone Positioning (Yes)	25.0% (3/12)	28.6% (2/7)	0.0% (0/5)	0.656
Inhaled Nitric Oxide (Yes)	0.0% (0/12)	28.6% (2/7)	0.0% (0/5)	0.112
Blood Transfusion (Yes)	8.3% (1/12)	0.0% (0/7)	0.0% (0/5)	1.000

For categorical variables, significance was tested using the Chi-squared test with Yates’s correction, or the exact Fisher test if any expected value was <5, and the percentage and fraction of patients fitting the category is displayed. For continuous variables, the Kruskal-Wallis test was used, and the mean ± standard deviation of the variable is displayed, with the number of patients assessed in brackets. Significant p-values (p <0.05) are bolded, indicating the metadata variable significantly differs across the three endotypes. Non-significant differences in comorbidities, symptoms, and follow-up metrics are in **Table S3**.

Gene expression trajectories differed dramatically between these three endotypes, particularly in inflammation and hemostasis activity. This was visualized by using gene set variation analysis (GSVA; a non-parametric unsupervised method of estimating gene set enrichment) to calculate enrichment scores of two Hallmark gene sets, “Inflammatory Response” and “Coagulation” (**Figure 2B**), which were of interest based on the trajectory responses of symptomatic and asymptomatic patients described above (**Figures 1C**, **S2B**). The Resolved endotype, with the lowest proportion of patients with post-COVID symptoms, had elevated enrichment of these mechanisms while hospitalized that significantly decreased after discharge, thus they “resolved” their inflammatory and hemostasis responses (**Figure 2B**). Conversely, the Suppressive endotype, with the highest proportion of patients with post-COVID symptoms, had low enrichment of the inflammatory and coagulation gene sets in hospital. Inflammation further decreased after discharge while coagulation increased slightly, although both still had relatively low enrichment. Consequently, these processes were considered “suppressed” (**Figure 2B**). Lastly, the Unresolved endotype, which also had a substantial proportion of patients with post-COVID symptoms, had persistently high enrichment of dysregulated inflammatory and coagulation gene sets in hospital that continued after discharge. Thus, these processes remained “unresolved” after discharge (**Figure 2B**). These inflammatory trajectories were also reflected in estimated neutrophil proportions, where the Resolved and Suppressive endotypes both significantly decreased over time, while the Unresolved endotype had persistently high neutrophil proportions (**Figure 2C**). Interestingly, the Suppressive endotype was the only endotype with a significant increase in regulatory T cell proportions over time, which might have contributed to persistent immune suppression (**Figure 2C**). These temporal patterns were recapitulated in the temporal patterns of inflammatory (*e.g.*, “Neutrophil degranulation”, “IL-1 signaling”) and hemostasis pathways (*e.g.*, “Platelet degranulation”, “Platelet activation, signaling, and aggregation”) when performing pathway enrichment of the DE genes over time for these three endotypes (**Figure S4A**).

**Figure 3:**
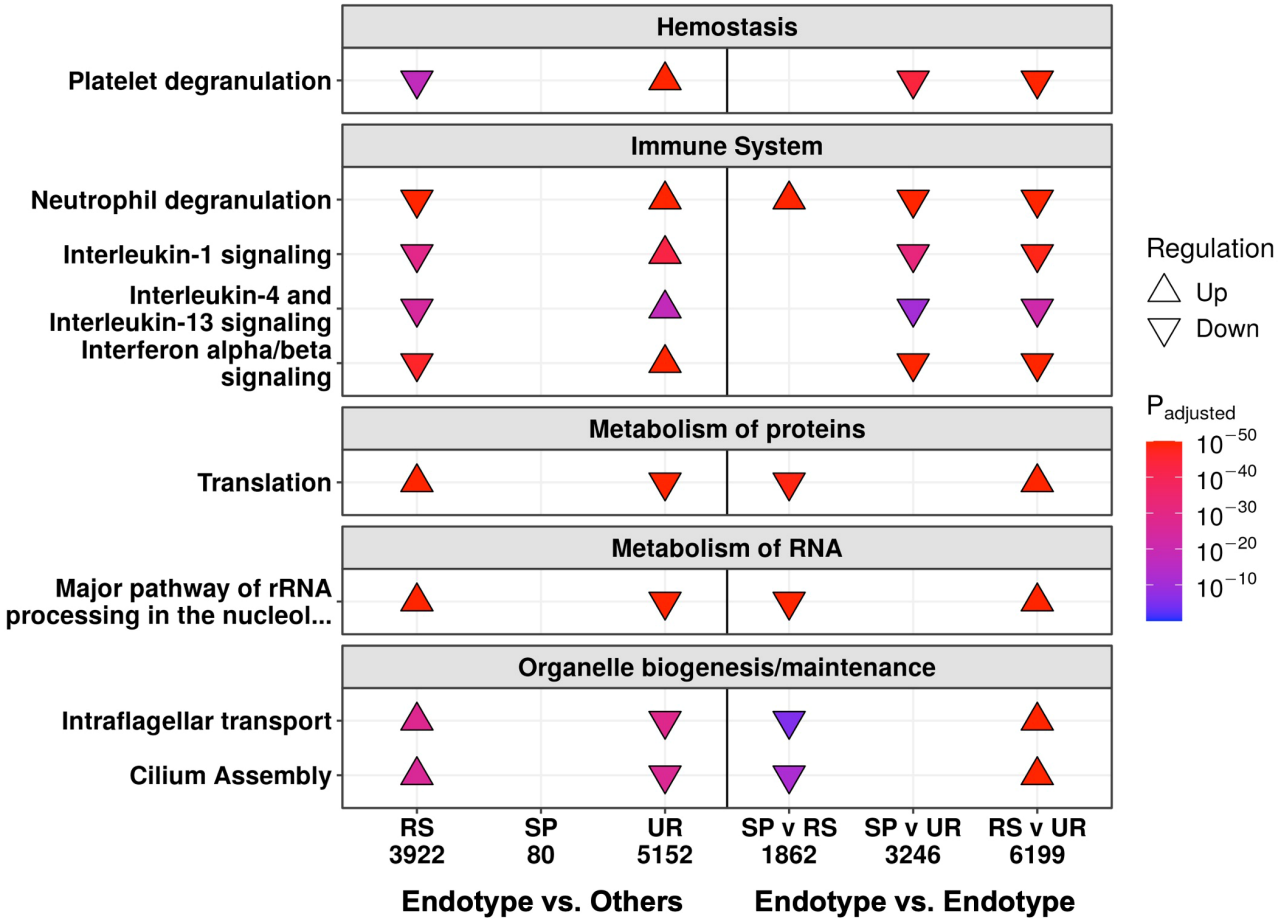
At follow-up, the Resolved (RS) and Unresolved (UR) endotypes had multiple oppositely regulated underlying mechanisms. Shown are a subset of the enriched Reactome pathways, with all enriched pathways in **Figure S3E**. On the left, each endotype is compared to the rest of the follow-up samples not in that endotype (*e.g.*, RS compared to Suppressive/SP and UR). On the right, each endotype is compared to another endotype, indicating that the SP endotype patients had cellular function changes like UR and immune changes like RS. The direction of arrows indicates whether the pathway was up- or down-regulated. The total number of DE genes are displayed under each comparison.

To further probe these mechanisms in more detail, pathway enrichment was performed on the DE genes between these three endotypes at follow-up and in-hospital. Follow-up samples in the Unresolved endotype had the most DE genes when compared to the samples in the other two endotypes (5,152 genes), followed by the Resolved endotype (3,922 genes), while the Suppressive endotype had only 80 DE genes (**Figure S3C**). Pathway enrichment using these DE genes showed that the Resolved and Unresolved endotypes were to some extent opposites of one other. The Resolved endotype had down-regulated hemostasis (“Platelet degranulation”) and immune pathways (“Neutrophil degranulation”, “IL-1 signaling”, “IL-4/13 signaling”, and “Interferon α/β signaling”), but upregulated cellular processes pathways (RNA processing, organelle biogenesis, and protein metabolism pathways) when compared to the rest of the samples, while the Unresolved endotype had the reverse (high immune/hemostasis, low cellular processes) (**Figure 3**, left). The 80 DE genes in the Suppressive endotype were enriched for only a single pathway (“Stimuli-sensing channels”) (**Figure S3E**). This low number of DE genes likely reflected the Suppressive endotype separately sharing certain mechanisms with each of the mechanistically distinct Resolved and Unresolved endotypes, thus diminishing differences when samples in the Suppressive endotype were compared to the samples in the other two endotypes. The endotype vs. endotype comparisons (*e.g.*, Suppressive vs. Resolved) were consistent with this suggestion. The Suppressive and Unresolved endotypes both had down-regulated cellular processes pathways when compared to the Resolved endotype (**Figure 3**, right). Conversely, the Suppressive and Resolved endotypes both had down-regulated hemostasis and immune pathways when compared to the Unresolved endotype (**Figure 3**, right), which was consistent with the GSVA enrichment scores at follow-up (**Figure 2B**).

We then determined how patients in these endotypes differed while in hospital. Only the Suppressive endotype had a substantial number of DE genes (2,963 genes) when compared to the other endotypes, while few or no DE genes were seen in the Resolved (0 genes) and Unresolved (18 genes) endotypes (**Figure S5A**). It appeared that hospital samples from the Resolved and Unresolved endotypes were quite similar to each other, since the direct comparison of hospital samples between these two endotypes yielded only 16 DE genes (**Figure S5B**), and on principal component analysis (an unsupervised clustering approach based on gene expression variation), these two endotypes overlapped while the Suppressive endotype clustered on its own (**Figure S5C**). Based on pathway enrichment results (**Figure S5D**), the Suppressive endotype was mainly differentiated from the other two endotypes in hospital by lower expression of genes involved in immune pathways such as “Neutrophil degranulation” and “Immunoregulatory interactions” and hemostasis pathways such as “Platelet degranulation” and “Platelet activation, signaling, and aggregation”, which was again consistent with the GSVA enrichment scores in-hospital (**Figure 2B**).

Overall, resolution of immune and hemostatic function (Resolved endotype) was associated with a lower rate of post-COVID symptoms, while persistently low (Suppressive) or high (Unresolved) immune and hemostatic function were associated with a higher rate of post-COVID symptoms.

### 3.5 Gene signatures could accurately distinguish the three endotypes

**Figure 4:**
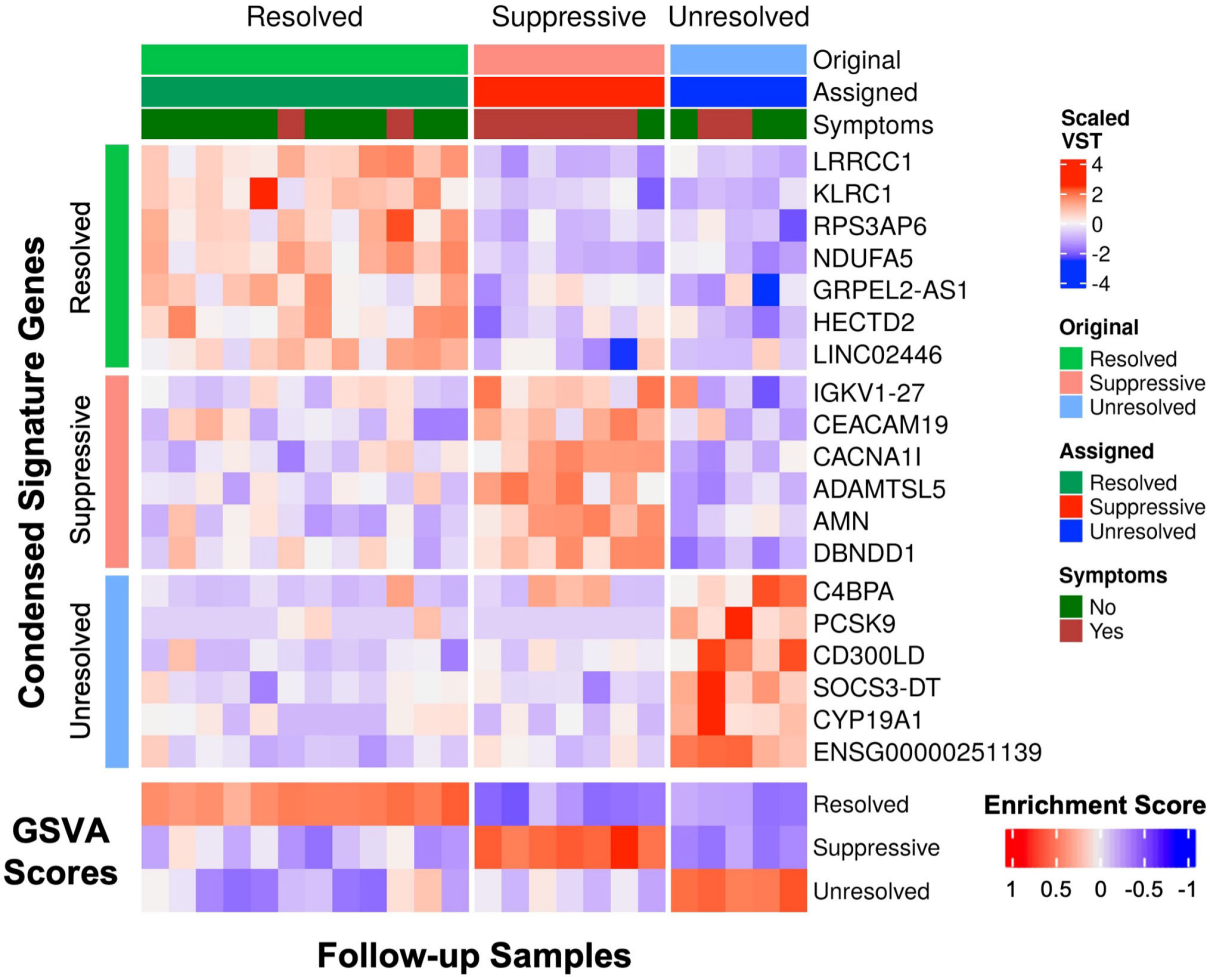
The condensed gene signatures accurately classified all follow-up samples into one of the three endotypes. The top heatmap displays scaled variance stabilized transformed (VST) counts of the condensed signature genes. The bottom heatmap displays the GSVA enrichment scores of each endotype for each follow-up sample. Samples were assigned to an endotype based on which endotype had the highest gene set variation analysis (GSVA) enrichment score (“Assigned”). The assigned endotypes were identical to the original annotated endotype from K-medoids clustering (“Original”), indicating perfect classification of these samples using these condensed gene signatures.

Specific gene signatures differentiating the three endotypes were identified for potential use in diagnosis or guiding treatment of patients at follow-up. The most significantly upregulated genes from each endotype (top 38 for Suppressive, top 50 for Resolved and Unresolved) relative to all other endotypes at follow-up were used as a preliminary gene expression signature (**Table S4**). Using GSVA, patients were assigned an endotype based on the relative enrichment of these signatures, and only one patient was misclassified (**Figure S6A**). We then determined if this gene signature could be condensed into a smaller number of genes to make simpler and more generalizable gene signatures. Using Least absolute shrinkage and selection operator (LASSO) regression (42), which can eliminate less influential predictor variables (genes) using 10-fold cross-validation, the gene signatures for each endotype were reduced to 6-7 genes for the Resolved (*LRRCC1*, *KLRC1*, *RPS3AP6*, *NDUFA5*, *GRPEL2-AS1*, *HECTD2*, *LINC02446*), Suppressive (*IGKV1-27*, *CEACAM19*, *CACNA1I*, *ADAMTSL5*, *AMN*, *DBNDD1*), and Unresolved (*PCSK9*, *C4BPA*, *CD300LD*, *SOCS3-DT*, *CYP19A1*, *ENSG00000251139*) endotypes. Classification of samples using GSVA with the condensed gene signature resulted in perfect classification with 100% accuracy (**Figure 4**).

To confirm the presence of endotypes in follow-up patients, we reanalyzed a publicly-available dataset on a cohort of 65 discharged COVID-19 patients with blood collected at 12, 16, or 24 weeks post infection onset (GSE169687) (24). The signatures were again able to classify patients in this validation cohort into three distinct endotypes (**Figure S6B**) with differing immune and hemostasis function (**Figure S6C**). The patients classified in the Unresolved endotype again had significantly higher enrichment of the Inflammatory and Coagulation gene sets compared to the Resolved and Suppressive endotypes (**Figure S6C**), while the Suppressive endotype was again an “intermediate” endotype clustering between the Resolved and Unresolved endotypes (**Figure S6D**), consistent with analyses of our cohort (**Figures 2A, B**).

## 4 Discussion

To better understand the pathophysiology underlying persistent post-COVID symptoms, gene expression differences were analyzed by comparing COVID-19 survivors who reported having persistent symptoms or not 4-12 weeks after discharge. Interestingly, cross-sectional comparison of patients with and without symptoms at follow-up and in hospital yielded almost no DE genes (**Figures S1D, E**), which was likely primarily related to the presence of three gene expression clusters, or endotypes, as well as other confounders contributing to heterogeneity that confounded direct comparisons.

In contrast, trajectory analysis, which accounts for individual factors including genetics, demographics, diet, microbiome, and age, highlighted how patients with and without post-COVID symptoms differed over time. Asymptomatic patients at follow-up showed massive gene expression changes related in part to a decrease, relative to their time in hospital, in clotting pathways and inflammation (**Figures 1A, C**). These are two key processes that have been highlighted to play a role in acute COVID-19 pathogenesis (50) and potentially in long COVID. In addition, there was an increase in the activity of adaptive immunity pathways, which may be reflective of a recovering adaptive immune response, since decreased adaptive immunity is also associated with poor outcomes in acute COVID-19 (54). The lack of these changes in symptomatic patients (**Figure 1C**) indirectly suggested that these processes may stay dysregulated in these patients and might potentially contribute to the development of persistent symptoms. Individual studies have indeed indicated persistent T cell functional deficits (55–57), inflammation (15, 16), and clotting abnormalities (23) in patients with post-COVID symptoms but have not looked at this issue in a temporal manner based on whole blood gene expression described in this study.

As an alternative strategy to investigating the impact of heterogeneity, based on underlying disease processes, unsupervised machine learning was performed to separate patients based on gene expression differences (*i.e.*, mechanistic endotypes), rather than the presence or absence of symptoms. Endotypes have been used to study heterogeneity in both sepsis and COVID-19 (41) and were proposed to be present based on clinical/disease features in a long-COVID cohort (49). Here, patients were separated into three endotypes, based on mechanistically-linked gene expression differences. Each endotype had substantially varying proportions of post-COVID symptoms: Resolved (almost all patients were asymptomatic), Suppressive (almost all patients were symptomatic), and Unresolved (some symptomatic patients) (**Figure 2A**; **Table 2**), with specific gene expression biomarkers for each (**Figure 4**) that could distinguish endotypes with high and low rates of post-COVID symptoms; in contrast, clinical risk factors such as sex and smoking failed to distinguish endotypes in this cohort (**Table 1**).

Two key pathway groups stood out as differentiating the three endotypes: inflammatory (*e.g.*, interleukin, interferon) and hemostasis (*e.g.*, platelet degranulation) pathways (**Figure 2B**). The Resolved endotype had elevated expression of genes involved in inflammation and hemostasis pathways that decreased by discharge, suggesting resolution of these processes. The Suppressive endotype had low expression of inflammation and hemostasis genes in hospital and at follow-up, while the Unresolved endotype had high expression of these genes both in hospital and at follow-up. Overall, these comparisons suggested that proper initiation and then resolution of such responses (Resolved endotype) was associated with a significantly lower incidence of developing post-COVID sequelae, while failure to modulate these responses (Suppressive and Unresolved endotypes) was associated with development of these symptoms. Intriguingly, the Resolved endotype had the lowest rates of systemic corticosteroid use when hospitalized, with approximately half (41.7%) receiving corticosteroids compared to almost all patients in the other endotypes (85.7% and 100%) (**Table 2**), which could be an avenue of further investigation. Exogenous steroids can suppress endogenous steroid production, which might be occurring in the Suppressive and Unresolved endotypes, disrupting immune function, and low endogenous steroid levels have been documented in patients with post-COVID symptoms in another cohort (49). Use of such corticosteroids in-hospital could potentially interfere with mounting and resolving an appropriate immune response during COVID-19 and might increase the risk of persistent symptoms after discharge.

The mechanistic differences between the three endotypes (that were recapitulated in a validation dataset, **Figure S6B**), suggested different pathophysiological reasons underlying the presence or absence of symptoms. Persistently low immune responses in the Suppressive endotype may impede clearance of the virus or enhance susceptibility to other infections or reactivation of latent infections such as Epstein-Barr virus, all of which may contribute to symptoms (22, 49). The increase in regulatory T cells may be contributing to the continually suppressed immune response observed in this endotype (**Figure 2C**). Low hemostatic function has also been associated with patients referred to a long-COVID clinic (24). Conversely, maintaining a high inflammatory and hemostatic response even after discharge is also likely to be detrimental, as seen in the Unresolved endotype (in which 40% or patients were symptomatic after discharge), potentially due to sustained autoimmune or inflammatory damage (18, 49), as well as microclot formation (23). The enrichment of interferon signaling pathways in the Unresolved endotype at follow-up (**Figure 3**) may also suggest ongoing viral infection as well. Overall, these hypotheses should be investigated further by analyzing the presence of viral RNA, auto-antibodies, and immune markers in follow-up patients in conjunction with gene expression in a future, larger study.

There are some limitations to this study. These findings are from a small cohort, in part due to logistic difficulties in obtaining high numbers of follow-up patients early in the pandemic, although we also observed the new endotypes in another public dataset. These patients were also unvaccinated and infected with earlier COVID-19 variants, thus future studies could evaluate these endotypes in vaccinated populations infected by current variants. Symptoms were self-reported, which could potentially add a degree of subjectivity to the analysis; this was difficult to mitigate for subjective symptoms such as fatigue. In addition, further subgrouping of patients based on specific symptoms in this cohort was not feasible due to the sample size, which is why patients were only separated into those with and without post-COVID symptoms. A larger future cohort might further elucidate whether there are specific trajectories associated with distinct inflammatory and coagulation pathways. Lastly, this analysis was performed on whole blood, and therefore symptoms that were localized to the brain, lung, or muscle, might not be easily detected in the blood. Nevertheless, a simple blood test is more convenient and clinically safer than an invasive tissue biopsy. Thus, the findings in this study can facilitate the development of a clinical gene biomarker panel (*e.g.*, further clinical validation with quantitative real-time PCR) with the gene expression signatures for each endotype identified in the study (**Figure 4**) to better understand and potentially treat the underlying personalized pathophysiology of each patient. Future studies could investigate these endotypes at even later time points.

In conclusion, failure to modulate inflammatory and hemostatic pathways was associated with a higher rate of development of persistent symptoms after hospitalization for a COVID-19 lung infection, and vice versa, suggesting that a dynamic immune and coagulation system was protective against “long COVID”. Modulating these processes in patients suffering from persistent post-COVID symptoms, guided by gene expression signatures to determine whether to dampen a persistently activated response or boost a lacklustre response to restore homeostasis, may be essential to ensure that these patients can return to a better quality of life after COVID-19.

## Data availability statement

The datasets presented in this study can be found in online repositories. The names of the repository/repositories and accession number(s) can be found below: GSE221234 and GSE222253 (GEO). Code is available upon request (RH, bob@hancocklab.com).

## Ethics statement

The studies involving humans were approved by the Clinical Research Ethics Board of the University of British Columbia and Comité d’éthique de la recherche du Centre hospitalier de l’Université de Montréal. The studies were conducted in accordance with the local legislation and institutional requirements. The participants provided their written informed consent to participate in this study.

## Author contributions

RH and RL conceived the study. AA, AB, and RH contributed to the study design. AA performed bio-informatics analysis and wrote the initial draft of the paper. AA, AB, PZ, TB, JG, AL, and RH contributed to interpretation of data. DK and RL coordinated and were directly involved in sample and patient metadata collection in hospitals. AA, AB, TB, EA, PZ, and AL verified the quality and accuracy of sequencing and clinical data. RH, AL, and RL were responsible for obtaining funding. RH led the study and extensively edited the manuscript. All authors contributed to the article and approved the submitted version.
