# Supplementary material for: Post-COVID symptoms are associated with endotypes reflecting poor inflammatory and hemostatic modulation
Source: Front Immunol. 2023 Aug 23;14:1243689. doi: 10.3389/fimmu.2023.1243689 (PMC10482103; doi:10.3389/fimmu.2023.1243689)
Supplement: Supplementary file 1 [file DataSheet_1.docx]

Supplementary Material

Post-COVID symptoms are associated with endotypes reflecting poor inflammatory and hemostatic modulation

Andy Y. An^1^, Arjun Baghela^1^, Peter Zhang^1^, Travis Blimkie^1^, Jeff Gauthier^2^, Daniel E. Kaufmann^3,4^, Erica Acton^5^, Amy H.Y. Lee^5^, Roger C. Levesque^2^, Robert E.W. Hancock*^1^

^1^Centre for Microbial Diseases and Immunity Research, University of British Columbia, 232-2259 Lower Mall, Vancouver, BC, V6T 1Z4, Canada

^2^Département de microbiologie-infectiologie et d'immunologie, Université de Laval, Laval, Québec

^3^Department of Medicine, Université de Montréal, Montréal, Canada

^4^Fonds de recherche du Québec (FRQ) COVID-19 Biobank

^5^Department of Molecular Biology and Biochemistry, Simon Fraser University, 8888 University Drive, Burnaby, BC, V5A 1S6, Canada

*** Correspondence:** Dr. Robert EW Hancock: [bob@hancocklab.com](mailto:bob@hancocklab.com)

# Supplementary Figures


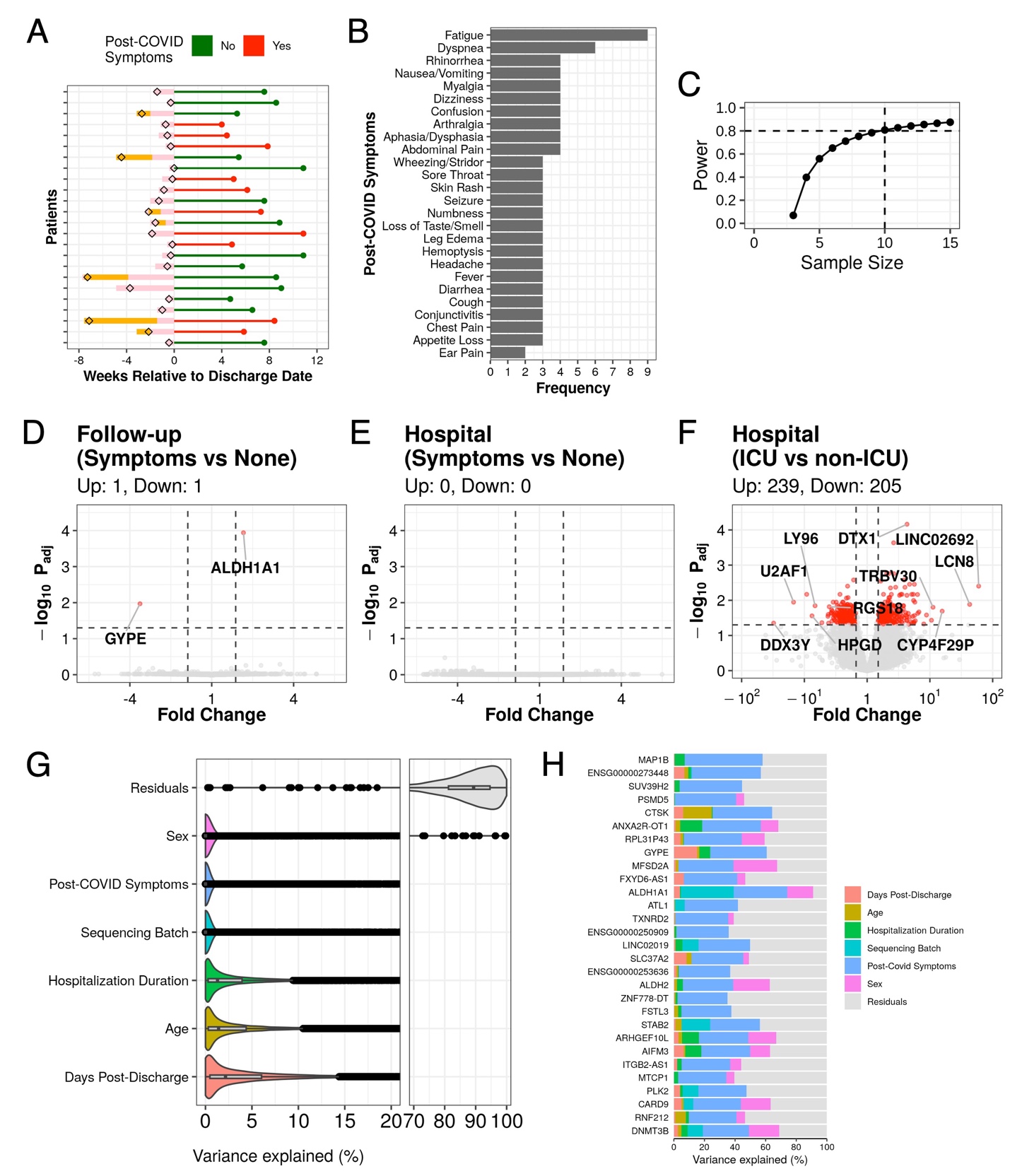


Figure S1. Timelines, reported symptoms, and comparisons between patients with and without post-COVID symptoms.

**A:** Hospitalization duration (orange = ICU, pink = non-ICU hospitalization) and follow-up visit time relative to discharge date. Open diamonds indicate the time of blood draw in-hospital, while the filled circles indicate time of blood draw at follow-up visit, with the colour corresponding to presence or absence of symptoms during the follow-up visit. **B:** Frequency of symptoms of the ten patients who self-reported persistent post-COVID symptoms at the follow-up visit. **C:** Power calculation for minimum sample size. To achieve a power of 0.8 with a false-discovery rate of 0.05 to detect 15% of the transcriptome to be differentially expressed, a minimum of 10 samples in one group was needed. This meant that the initial analysis comparing patients with post-COVID symptoms (10 patients) to those without (14 patients) was adequately powered. The gene dispersions for power calculations were estimated based on a published RNA-Seq dataset of patients with post-COVID-19 symptoms, GSE169687, and power estimation calculation was performed using the package *ssizeRNA*. **D, E, F:** Volcano plot of DE genes between different comparisons. Coloured dots indicate DE genes (adjusted p-value <0.05, absolute fold change ≥1.5). The top 5 up- and down- regulated genes (lowest adjusted p-value and highest fold change) are labelled. **D:** Two DE genes (*GYPE*, *ALDH1A1*) were identified between patients with post-COVID symptoms compared to those without post-COVID symptoms during the follow-up visit. **E:** No DE genes were identified between patients who later developed post-COVID symptoms compared to those who did not while they were hospitalized. **F:** 444 DE genes were identified between ICU and non-ICU patients during hospital sampling. **G:** Analysis of potential confounders influencing gene expression variation of follow-up samples. Percent variance of each gene explained by each metadata variable was calculated using the package *variancePartition*. Points indicate genes outside 1.5 times the interquartile range. Violin plots indicate median and interquartile ranges of percent variance of all genes for each variable. Most genes have variance attributed to none of the metadata variables listed and are instead attributed to “Residuals”; high residuals may indicate the presence of individual-specific differences (*e.g.*, genetic, underlying comorbidities, etc.). **H**: Percent variance explained by each metadata variable for 29 genes that have >30% of variance explained by the presence or absence of post-COVID symptoms. Of these genes, *GYPE* and *ALDH1A1* were differentially expressed between patients with and without post-COVID symptoms at follow-up (Panel D).

**
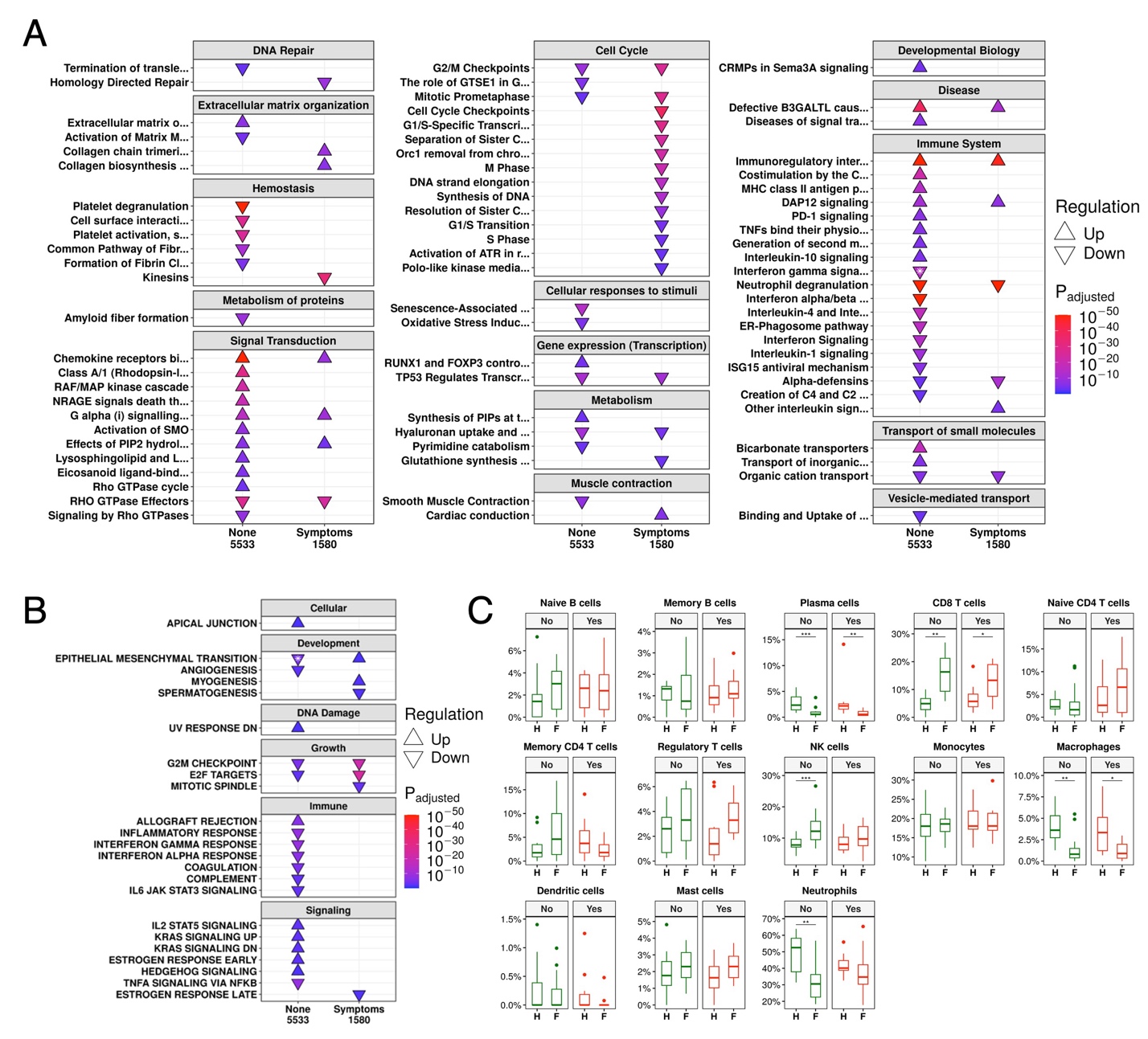
**

**Figure S2. Patients with and without post-COVID symptoms had substantially different trajectories of pathways and cell proportion changes, consistent with inflammatory and hemostasis resolution. A:** All enriched Reactome pathways from trajectory analysis (follow-up vs. in-hospital samples) of symptomatic and asymptomatic patients. A subset of enriched pathways is shown in **Figure 1**. Asymptomatic patients at discharge (“None”) had temporally enriched pathways consistent with immune resolution, which was not seen in patients with post-COVID-19 symptoms (“Symptoms”). For one pathway (“Interferon gamma signaling”), both directions were enriched (indicated by *, the direction with the lower adjusted p-value is shown). The total number of DE genes in each comparison are shown under each label. **B:** Enriched Hallmark gene sets using DE genes over time in asymptomatic (“None”) and symptomatic (“Symptoms”) patients. For one gene set (“Epithelial mesenchymal transition”), both directions were enriched (indicated by *, the direction with the lower adjusted p-value is shown). The total number of DE genes in each comparison is shown under each label. **C:** Estimated cell proportions of in-hospital and follow-up samples in patients with or without post-COVID symptoms after discharge. Cell proportions were estimated by *CIBERSORTx*. A pair-wise Wilcoxon rank-sum test between in-hospital and follow-up samples was performed to determine significance and adjusted for multiple corrections (Benjamini-Hochberg): *** = p<0.001, ** = p<0.01, and * = p<0.05. No: No symptoms, Yes: Symptomatic, H: Hospital samples, F: Follow-up samples.


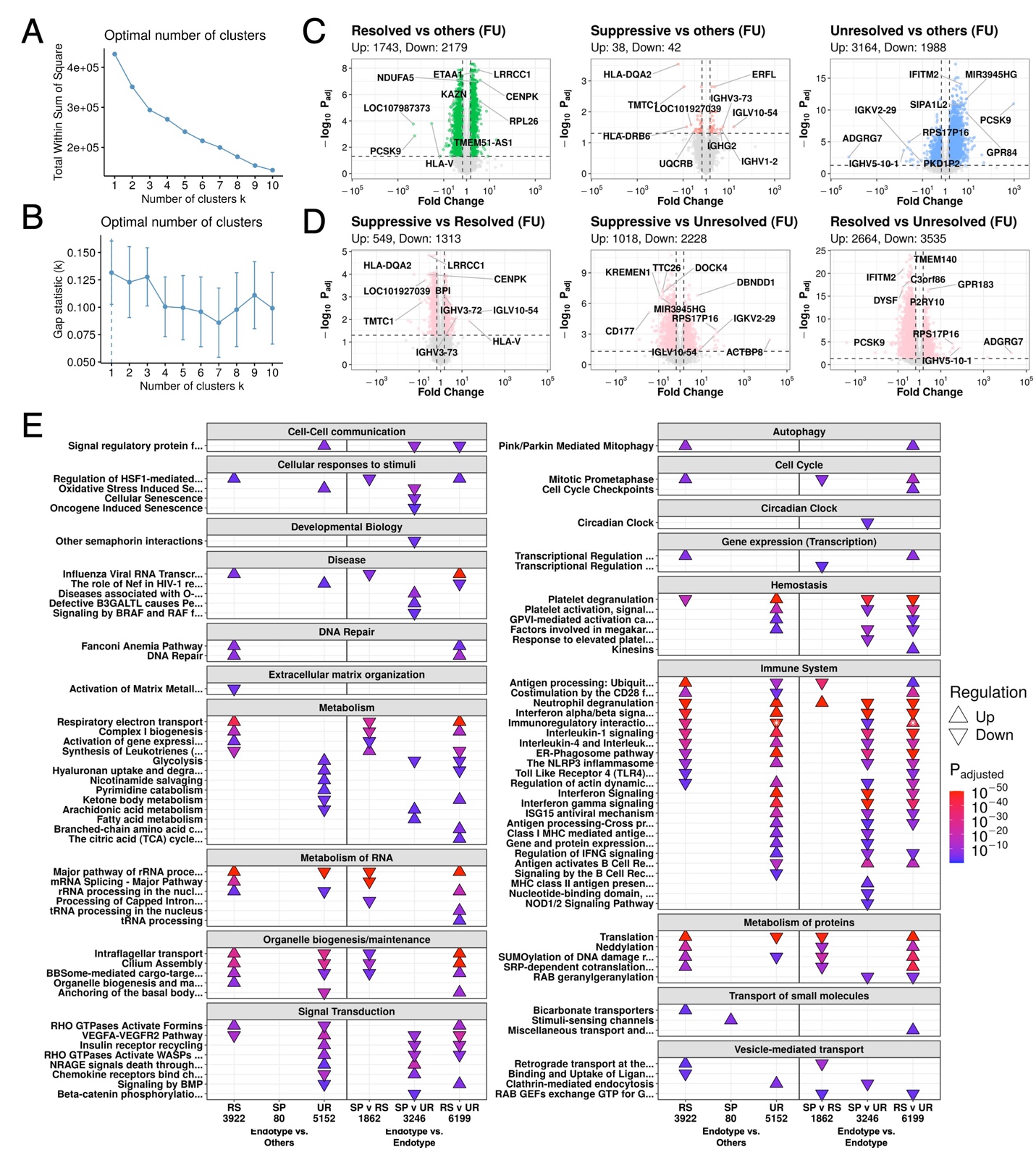
**Figure S3. Patients were separated into three endotypes with differing mechanisms at follow-up: Resolved, Suppressive, and Unresolved. A, B:** Clustering metrics using k-medoid clustering showed that the optimal cluster number was *k* = 3. **A**: Optimal number of clusters based off total within sum of square, where the optimal number of clusters is associated with the “elbow” of the graph. **B**: Optimal number of clusters based off the gap statistic, where the optimal number of clusters is associated with the highest gap statistic. Both total within sum of squares and gap statistic results indicated that three was the optimal number of clusters. **C:** Volcano plot of DE genes from follow-up samples of each endotype to the rest of the follow-up samples not in that endotype. **D:** Volcano plot of DE genes between each endotype to another endotype. Coloured dots indicated DE genes (p-adj <0.05, absolute fold change ≥1.5). The top 5 up- and down-regulated genes (lowest adjusted p-value and highest fold change) are labelled. FU = Follow-up. **E:** All enriched Reactome pathways from DE genes between endotypes are shown here, and a subset of enriched pathways is shown in **Figure 3**. On the left side of each graph, each endotype is compared to the rest of the follow-up samples not in that endotype, *e.g.*, Resolved (RS) compared to Suppressive (SP) and Unresolved (UR). On the right side of each graph, each endotype is compared to another endotype. The direction of arrows indicates whether the pathway was up- or down-regulated. For one pathway (“Immunoregulatory interactions”), both directions were enriched (indicated by *, the direction with the lower adjusted p-value is shown). The total number of DE genes in each comparison are shown under each label.

**
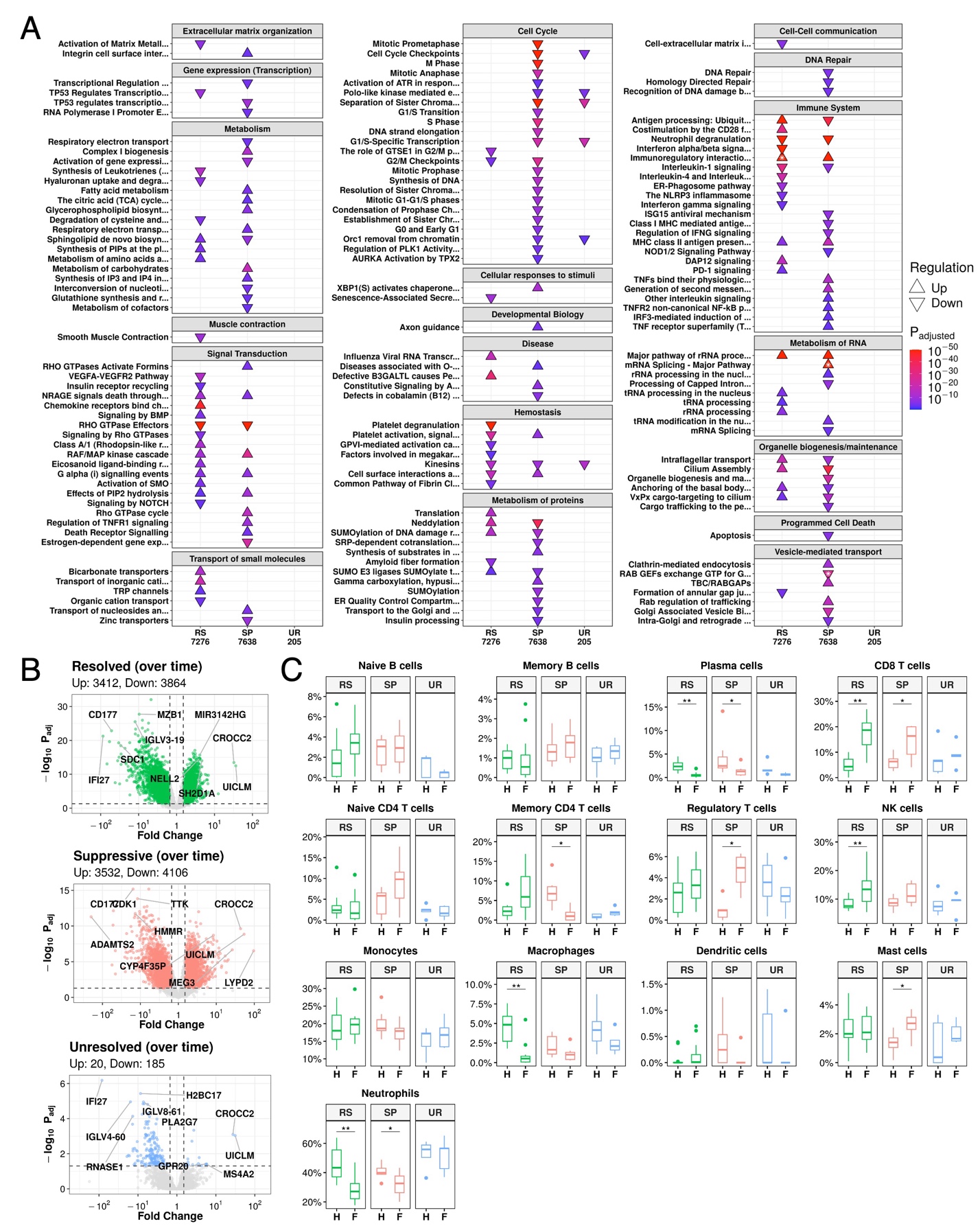
Figure S4.** **Gene expression trajectories were distinct between the three endotypes. A:** Enriched Reactome pathways using DE genes. For each endotype, the follow-up samples were compared to the hospital samples. The total number of DE genes in each comparison are shown under each label. For two pathways, both directions were enriched (indicated by *, the direction with the lower adjusted p-value is shown). RS = Resolved, SP = Suppressive, UR = Unresolved endotypes. The Resolved endotype (lowest proportion of symptomatic patients) demonstrated multiple inflammatory/innate-immune pathways that were all enriched by downregulated genes over time, while adaptive immune pathways such as “Costimulation by the CD28 family” were upregulated over time, much like what was observed in all asymptomatic patients (**Figure 1B**). For the Suppressive endotype (highest proportion of symptomatic patients), multiple cell cycle pathways were enriched by downregulated genes over time, consistent with what was seen in all symptomatic patients (**Figures 1B**). Some cell cycle pathways were also enriched in the Unresolved endotype, which also had a substantial proportion of symptomatic patients. Despite the Suppressive endotype having similar numbers of DE genes over time as the Resolved endotype, it did not enrich for many of the immune pathways seen in the Resolved endotype (“Interleukin-4/13 signaling”, “Interferon-γ signaling”, “Interferon-⍺/β signaling”, and “Co-stimulation by the CD28 family”). While the pathways “Neutrophil degranulation” and “Interleukin-1 signaling” were downregulated over time in both the Suppressive and Resolved endotypes, the Suppressive endotype already had low immune function in hospital when compared to the Resolved endotype (**Figures 2B, S5D**). Therefore, additional downregulation likely indicated further immune suppression over time in the Suppressive endotype. The Suppressive endotype also demonstrated upregulation over time of the hemostasis pathways “Platelet activation, signaling, and aggregation” and “Cell surface interactions at the vascular wall”, in contrast to the Resolved endotype where these two pathways were downregulated (resolved) over time. These opposing trends potentially signalled a convergence over time of hemostasis activity of these two endotypes. Specific genes involved are shown in **Figure S7**. **B:** Volcano plots of DE genes over time (between hospital and follow-up) for each endotype. Coloured dots indicated DE genes. For all plots, the top 5 up- and down-regulated genes (lowest adjusted p-value and highest fold change) are labelled. **C:** Cell proportions were estimated by *CIBERSORTx*. A pair-wise Wilcoxon rank-sum test between in-hospital and follow-up samples was performed to determine significance and adjusted for multiple corrections (Benjamini-Hochberg): *** = p<0.001, ** = p<0.01, and * = p<0.05. H: Hospital samples, F: Follow-up samples.


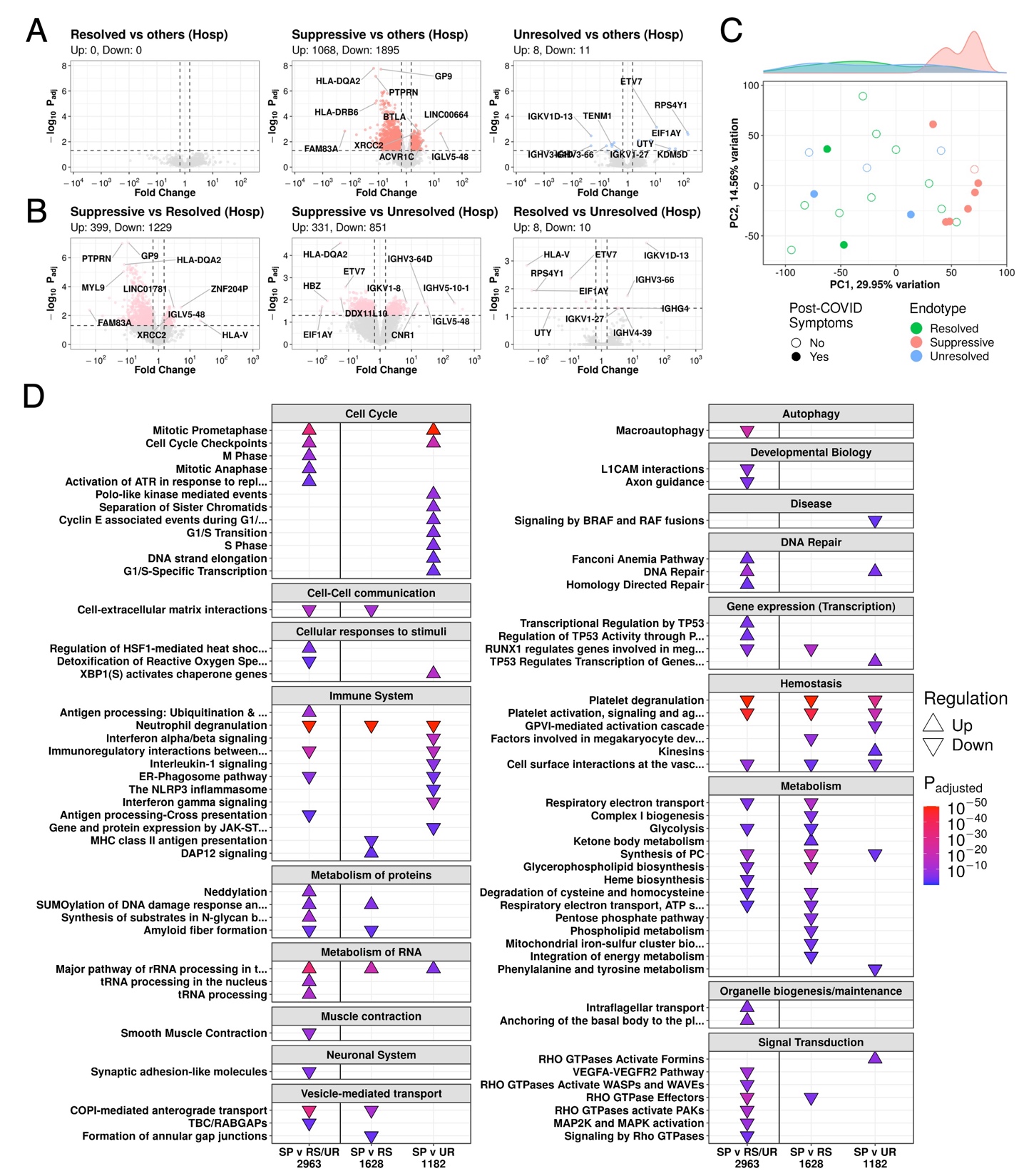


**Figure S5. In hospital, the Suppressive (SP) endotype was distinct from the Resolved (RS) and Unresolved (UR) endotypes. A:** Volcano plots of DE genes of hospital samples in each endotype compared to the rest of the hospital samples not in that endotype. **B:** Volcano plots of DE genes of hospital samples of each endotype compared to each other endotype. Coloured dots indicated DE genes. For all plots, the top 5 up- and down-regulated genes (lowest adjusted p-value and highest fold change) are labelled. **C:** Principal component analysis of hospital samples, coloured by endotype. The density plots on the top highlights the separation of samples from the Suppressive endotype from the Resolved and Unresolved endotypes (which substantially overlap) across the largest principal component PC1. **D:** Pathway enrichment of DE genes. Suppressive endotype hospital samples were compared to the other hospital samples not in that endotype (left side of graphs), and to Resolved and Unresolved endotypes individually (right side of graphs) to identify DE genes. The total number of DE genes in each comparison are shown under each label. No pathways were enriched from DE genes of Resolved vs others (0 DE genes), Unresolved vs others (19 DE genes), and Resolved vs Unresolved (18 DE genes) endotypes, and thus were not displayed.

**
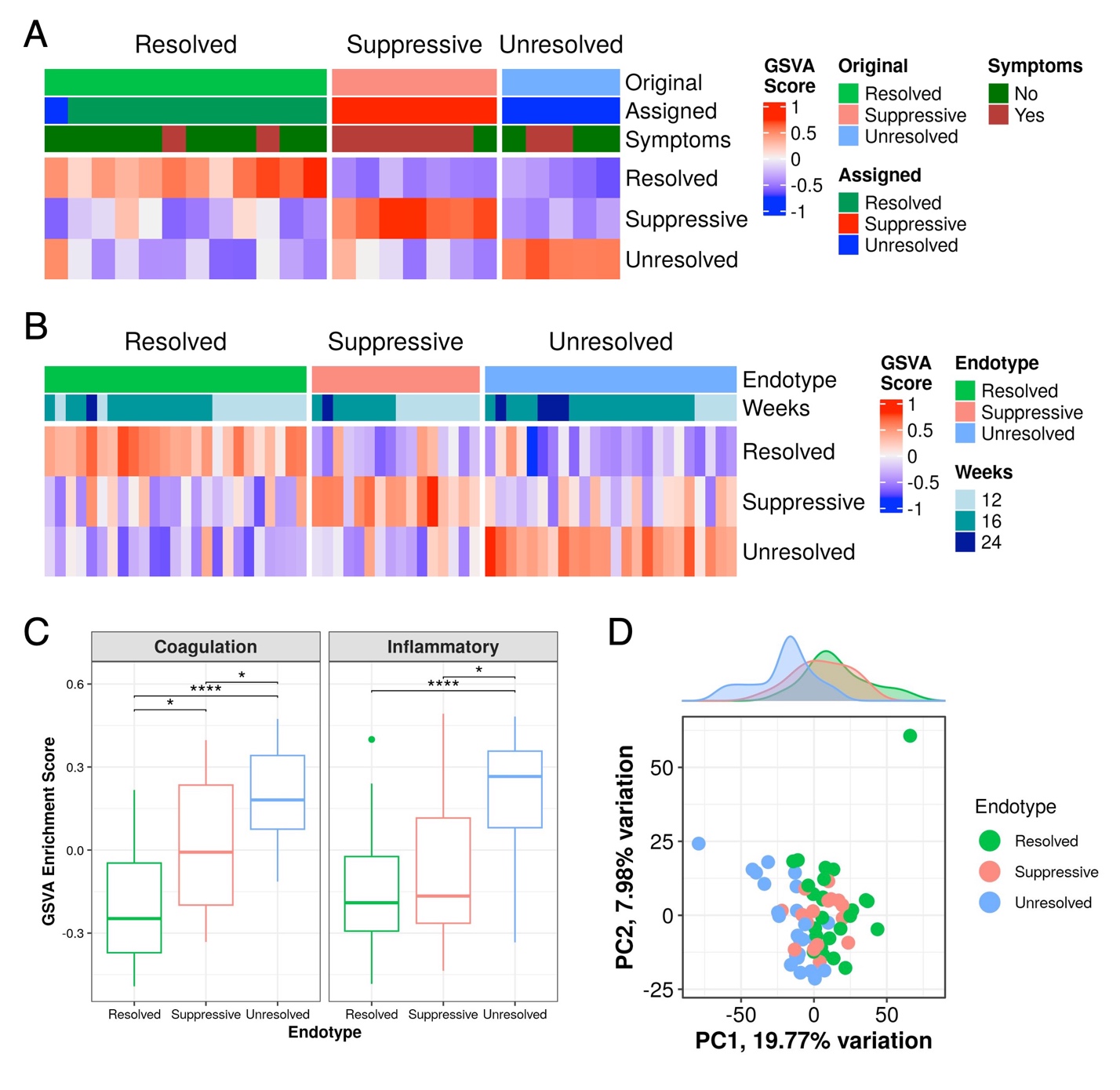
**

**Figure S6. Gene signatures for each endotype were developed and validated in an external cohort. A:** Heatmap displaying GSVA enrichment scores for each follow-up sample in this cohort using a preliminary signature of the top 38-50 upregulated genes in the three endotypes (**Table S4**). Each follow-up sample was assigned an endotype based on which endotype had the highest enrichment score (“Assigned”), and all but one sample were classified identical to the original annotated endotype from k-medoids clustering (“Original”). **B, C, D**: Endotypes were validated in post-COVID-19 samples from the publicly available dataset GSE169687. 65 patients had a blood sample collected at 12-, 16-, or 24-weeks post infection. **B:** Heatmap displaying gene set variation analysis (GSVA) enrichment scores using the condensed gene signature. Each patient was assigned an endotype based on which endotype had the highest enrichment score. **C:** GSVA enrichment scores of the Coagulation and Inflammatory Hallmark gene sets for the follow-up samples in this validation dataset that were assigned to each endotype (Wilcox ranked-sum test, * = p<0.05, **** = p<0.0001). **D:** PCA showed similar separation of three endotypes as the clustering patterns observed in the original samples (**Figure 2A**), with the Suppressive endotype being overlapping between the Resolved and Unresolved endotypes, which were more separate. The density plots on the top highlights the separation of samples across the largest principal component PC1.


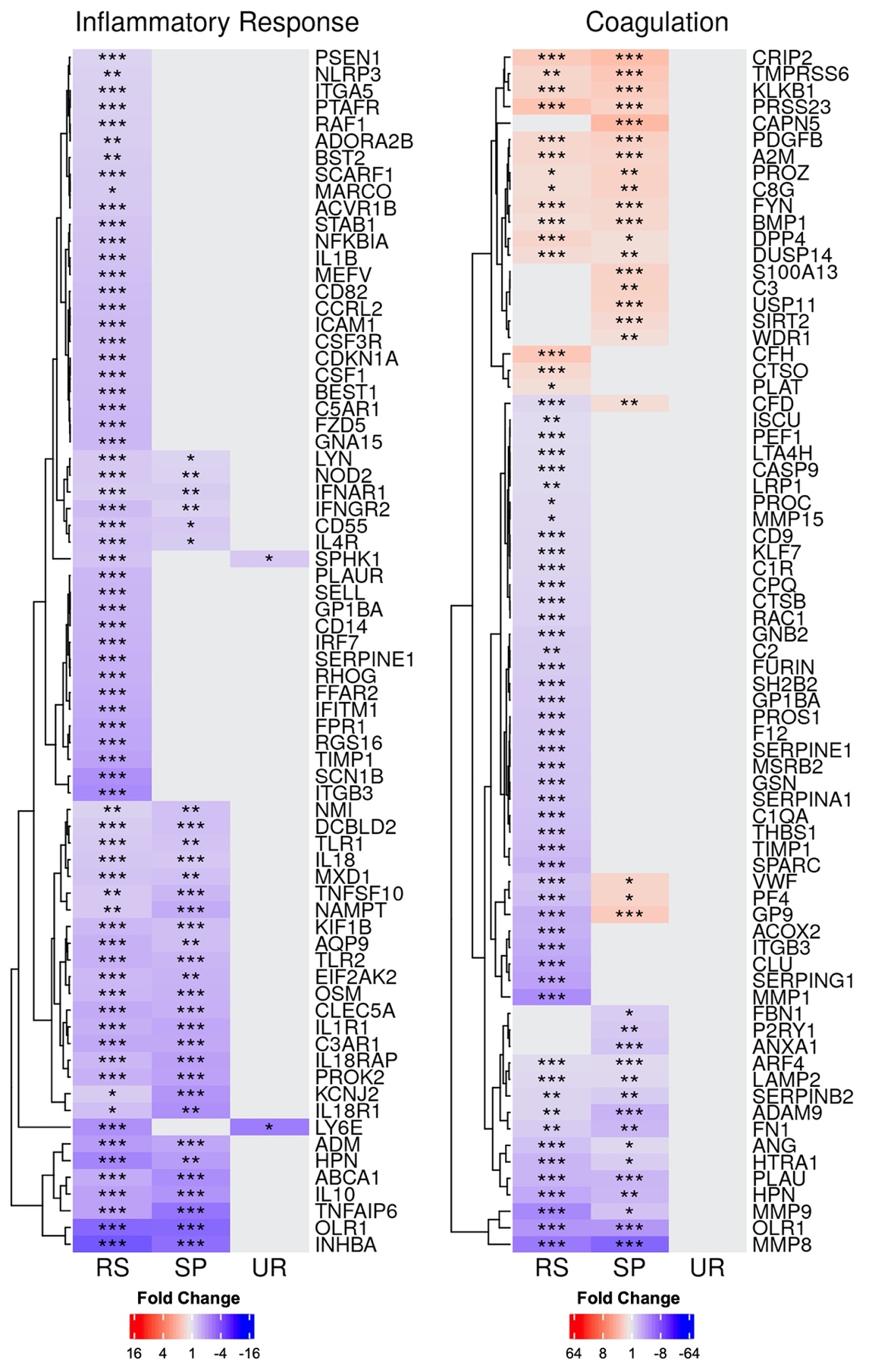


**Figure S7. Fold change heatmaps of differentially expressed (DE) genes over time involved in the Inflammatory Response and Coagulation gene sets are consistent with overall GSVA trends and pathway enrichment results.** For DE genes in the Inflammatory Response gene set, the Resolved (RS) and Suppressive (SP) endotypes had a substantial number of downregulated DE genes (including key inflammatory genes such as *ILR1*, *IFNGR2*, and *TLR1*), with more downregulated genes in the RS endotype. This was consistent with the trend of significant decreases over time in the Inflammatory Response gene set enrichment scores for the RS and SP endotypes (greater decrease in the RS endotype) observed in the GSVA analyses (**Figure 2B**). This was also consistent with significant downregulation over time of inflammation-related Reactome pathways such as “IL-1 signaling” and “Neutrophil degranulation” in the RS and SP endotypes (**Figure S4**). Likewise, for the DE genes in the Coagulation gene set, the RS endotype had a substantial number of downregulated DE genes, while the SP endotype had fewer downregulated DE genes and more upregulated DE genes (particularly clotting genes such as *VWF*, *PF4*, and *GP9*). This was consistent with the significant decrease over time of the Coagulation gene set enrichment score in the RS endotype and increase over time in the SP endotype (**Figure 2B**), as well as the downregulation over time of the clotting-related Reactome pathway “Platelet degranulation” in the RS endotype and upregulation over time of the Reactome pathway “Platelet activation, signaling, and aggregation” in the SP endotype (**Figure S4**). The Unresolved (UR) endotype had few DE genes over time, consistent with the lack of change over time of gene set enrichment scores and lack of significant enrichment of related Reactome pathways. Shading indicates the fold change over time (hospital to follow-up); only significant fold changes (adjusted P-value <0.05) are shown. Adjusted P-values were calculated from the DESeq2 model: * = p<0.05, ** = p<0.01, *** = p<0.001.

**Supplemental Tables**

**Table S1. Scoring Questionnaire of Follow-up Metrics.** As part of the questionnaire, patients recorded on a scale of 0 to 4 any difficulties in mobility, self-care, and performing activities of daily living, as well as presence of pain, anxiety/depression, and breathlessness. They also recorded on a scale of 0 to 2 any difficulties in various actions as well as frequency of falls. The total “Frailty score” is the sum of all these scores. Lastly, patients recorded on a scale of 0 to 100 how their health was on the day of follow-up, with 100 being the best health they could imagine and 0 being the worst. The “Poor Health Self-Rating” score was the inverse of this score (100 minus the patient’s self-reported score).

| **Metric** | **0** | **1** | **2** | **3** | **4** |
| --- | --- | --- | --- | --- | --- |
| Mobility Score | I have no problems in walking about | I have slight problems in walking about | I have moderate problems in walking about | I have severe problems in walking about | I am unable to walk about |
| Self-Care Score | I have no problem washing or dressing myself | I have slight problem washing or dressing myself | I have moderate problems washing or dressing myself | I have moderate problems washing or dressing myself | I have severe problems washing or dressing myself |
| Usual Activity Score | I have no problems doing my usual activities | I have slight problems doing my usual activities | I have moderate problems doing my usual activities | I have severe problems doing my usual activities | I am unable to do my usual activities |
| Pain and Discomfort Score | I have no pain or discomfort | I have slight pain or discomfort | I have moderate pain or discomfort | I have severe pain or discomfort | I have extreme pain or discomfort |
| Anxiety and Depression Score | I am not anxious or depressed | I am slightly anxious or depressed | I am moderately anxious or depressed | I am severely anxious or depressed | I am extremely anxious or depressed |
| Breathlessness Score | I am breathless only with strenuous exercise | I am short of breath when hurrying or going up a slight hill | I am slower than most people of the same age on level ground | I stop for breath walking 100m or few minutes on level ground | I am too breathless to leave the house |
| Difficulty carrying 10 lbs | None | Some | A lot or unable |  |  |
| Difficulty walking across a room | None | Some | A lot or unable |  |  |
| Difficulty climbing a flight of 10 stairs | None | Some | A lot or unable |  |  |
| Difficulty transferring from chair to bed | None | Some | A lot or unable |  |  |
| How many falls in the past year | None | 1-3 falls | 4 or more falls |  |  |

**Table S2.** **Patient demographics of patients with and without persistent post-COVID symptoms.** For categorical variables, significance was tested using the Chi-squared test with Yates’s correction, or the exact Fisher test if any expected value was <5, and the percentage and fraction of patients fitting the category is displayed. For continuous variables, the Wilcoxon Rank-Sum test was used, and the mean ± standard deviation of the variable is displayed, with the number of patients assessed in brackets. Follow-up metric scores are discussed in more detail in **Table S1**. Bolded P-values indicate significant differences (p <0.05).

| **Clinical Variables** | **No Post-COVID Symptoms (14)** | **Post-COVID Symptoms (10)** | **P-value** |
| --- | --- | --- | --- |
| Age | 60.1 ± 12.2 (14) | 54.5 ± 12.5 (10) | 0.306 |
| Sex (Male) | 85.7% (12/14) | 50.0% (5/10) | 0.085 |
| Body Mass Index | 30.6 ± 6.5 (12) | 28.7 ± 6.9 (7) | 0.526 |
| Admitted to ICU (Yes) | 28.6% (4/14) | 30.0% (3/10) | 1.000 |
| Smoker (Yes) | 25.0% (3/12) | 0.0% (0/7) | 0.263 |
| **Comorbidities** | | | |
| Asthma (Yes) | 14.3% (2/14) | 10.0% (1/10) | 1.000 |
| COPD (Yes) | 0.0% (0/14) | 10.0% (1/10) | 0.417 |
| Chronic Lung Disease (Yes) | 21.4% (3/14) | 10.0% (1/10) | 0.615 |
| Atrial Fibrillation/Flutter (Yes) | 14.3% (2/14) | 0.0% (0/10) | 0.493 |
| Hypertension (Yes) | 64.3% (9/14) | 10.0% (1/10) | **0.013** |
| Heart Failure (Yes) | 7.1% (1/14) | 0.0% (0/10) | 1.000 |
| Coronary Artery Disease (Yes) | 21.4% (3/14) | 0.0% (0/10) | 0.239 |
| Chronic Cardiac Disease (Yes) | 7.1% (1/14) | 20.0% (2/10) | 0.550 |
| Liver disease (Yes) | 14.3% (2/14) | 10.0% (1/10) | 1.000 |
| Chronic Hematologic Disease (Yes) | 0.0% (0/14) | 10.0% (1/10) | 0.417 |
| Chronic Kidney Disease (Yes) | 28.6% (4/14) | 0.0% (0/10) | 0.114 |
| Psychiatric Disease (Yes) | 21.4% (3/14) | 0.0% (0/10) | 0.239 |
| Rheumatologic Disease (Yes) | 0.0% (0/14) | 0.0% (0/10) | 1.000 |
| Obesity (Yes) | 21.4% (3/14) | 10.0% (1/10) | 0.615 |
| Diabetes (Yes) | 50.0% (7/14) | 0.0% (0/10) | **0.019** |
| AIDS (Yes) | 7.1% (1/14) | 0.0% (0/10) | 1.000 |
| Immunosuppressed (Yes) | 21.4% (3/14) | 10.0% (1/10) | 0.615 |
| Cancer (Yes) | 7.1% (1/14) | 20.0% (2/10) | 0.550 |
| **Arrival Values** | | | |
| Temperature (Celsius) | 37.2 ± 0.5 (14) | 37.1 ± 1.2 (10) | 0.557 |
| Systolic BP (mmHg) | 129.4 ± 15 (14) | 121.6 ± 19.1 (10) | 0.120 |
| Diastolic BP (mmHg) | 75.9 ± 11.3 (14) | 74.2 ± 8.2 (10) | 0.578 |
| Respiratory Rate | 21.3 ± 2.5 (14) | 24.6 ± 5.7 (10) | 0.095 |
| Heart Rate | 89.9 ± 12.8 (14) | 95.4 ± 21.5 (10) | 0.395 |
| O2 Saturation at Room Air | 95.8 ± 2.9 (9) | 94.4 ± 1.9 (7) | 0.363 |
| **Symptoms at Admission** | | | |
| Asymptomatic (Yes) | 7.1% (1/14) | 0.0% (0/10) | 1.000 |
| Confusion (Yes) | 0.0% (0/13) | 11.1% (1/9) | 0.409 |
| Diarrhea (Yes) | 28.6% (4/14) | 22.2% (2/9) | 1.000 |
| Abdominal Pain (Yes) | 7.7% (1/13) | 0.0% (0/9) | 1.000 |
| Chest Pain (Yes) | 28.6% (4/14) | 33.3% (3/9) | 1.000 |
| Dyspnea (Yes) | 71.4% (10/14) | 90.0% (9/10) | 0.358 |
| Dizziness (Yes) | 7.1% (1/14) | 11.1% (1/9) | 1.000 |
| Extremity Numbness (Yes) | 0.0% (0/13) | 22.2% (2/9) | 0.156 |
| Fatigue (Yes) | 42.9% (6/14) | 44.4% (4/9) | 1.000 |
| Fever (Yes) | 78.6% (11/14) | 55.6% (5/9) | 0.363 |
| Hemoptysis (Yes) | 0.0% (0/14) | 22.2% (2/9) | 0.142 |
| Loss of Appetite (Yes) | 21.4% (3/14) | 11.1% (1/9) | 1.000 |
| Sore Throat (Yes) | 7.1% (1/14) | 20.0% (2/10) | 0.550 |
| Headache (Yes) | 21.4% (3/14) | 10.0% (1/10) | 0.615 |
| Myalgia (Yes) | 21.4% (3/14) | 10.0% (1/10) | 0.615 |
| Nausea/Vomiting (Yes) | 21.4% (3/14) | 30.0% (3/10) | 0.665 |
| Loss of Taste/Smell (Yes) | 14.3% (2/14) | 10.0% (1/10) | 1.000 |
| Rhinorrhea (Yes) | 7.1% (1/14) | 10.0% (1/10) | 1.000 |
| Cough (Yes) | 57.1% (8/14) | 80.0% (8/10) | 0.388 |
| Aphasia/Dysphasia (Yes) | 0.0% (0/14) | 0.0% (0/10) | 1.000 |
| **Worst Laboratory Values** | | | |
| Highest %Neutrophil | 80.3 ± 7.9 (14) | 77.0 ± 8.0 (10) | 0.279 |
| Lowest %Lymphocyte | 6.8 ± 3.7 (14) | 6.8 ± 2.9 (10) | 0.838 |
| Highest %Monocyte | 8.6 ± 2.6 (14) | 9.2 ± 1.7 (10) | 0.364 |
| Lowest Platelets (10^3^ platelets/µL) | 244.4 ± 120.9 (14) | 228.4 ± 100.3 (10) | 0.884 |
| Lowest Hemoglobin (g/L) | 112.4 ± 30.9 (14) | 119.7 ± 23.6 (10) | 0.618 |
| Highest Urea (mg/dL) | 9 ± 3.7 (11) | 9.9 ± 7.2 (8) | 0.934 |
| Highest Creatinine (µmol/L) | 87.3 ± 37.9 (14) | 109.9 ± 142.1 (10) | 0.151 |
| Highest Sodium (mmol/L) | 141.9 ± 5.1 (14) | 143.8 ± 5 (10) | 0.215 |
| Highest Potassium (mmol/L) | 10.8 ± 20.3 (14) | 8.8 ± 13.1 (10) | 0.332 |
| Highest AST (units/L) | 59.6 ± 31 (11) | 94.2 ± 102.3 (5) | 0.777 |
| Highest ALT (units/L) | 51.6 ± 23.8 (14) | 70.8 ± 63.3 (9) | 0.614 |
| Highest Total Bilirubin (µmol/L) | 14.0 ± 10 (14) | 14.3 ± 12.8 (9) | 0.800 |
| Highest Glucose (mmol/L) | 15.2 ± 8.1 (14) | 9.9 ± 3.8 (9) | 0.166 |
| Highest Venous Lactate (mmol/L) | 1.9 ± 1.3 (6) | 1.9 ± 0.9 (5) | 1.000 |
| Highest D-Dimer (ng/mL) | 2977 ± 3769 (10) | 3363 ± 4142 (6) | 0.551 |
| Highest Fibrinogen (g/L) | 7.9 ± 2.4 (7) | 8.4 ± 1.5 (5) | 0.745 |
| Highest Ferritin (ng/mL) | 537.6 ± 536 (9) | 359.7 ± 296.9 (3) | 0.712 |
| Highest C-Reactive Protein (mg/L) | 155.5 ± 87.3 (14) | 128 ± 94.8 (10) | 0.429 |
| Highest LDH (units/L) | 341.2 ± 55.4 (13) | 336.4 ± 145.5 (5) | 0.324 |
| Highest Procalcitonin (ng/mL) | 0.2 ± 0.1 (11) | 0.6 ± 1.3 (6) | 0.480 |
| Highest Estimated SOFA Score | 3.3 ± 2.6 (14) | 4.1 ± 3.1 (10) | 0.456 |
| Highest WHO COVID-19 Score | 5.6 ± 1.4 (14) | 6 ± 1.4 (10) | 0.360 |
| Hospitalized Duration (Days) | 16.4 ± 14.8 (14) | 14.9 ± 14.5 (10) | 0.907 |
| **Treatments During Hospitalization** | | | |
| Antifungal (Yes) | 7.1% (1/14) | 10.0% (1/10) | 1.000 |
| Antibiotics (Yes) | 78.6% (11/14) | 80.0% (8/10) | 1.000 |
| Antivirals |  |  |  |
| Lopinavir/Ritonavir (Yes) | 21.4% (3/14) | 10.0% (1/10) | 0.615 |
| Remdesivir (Yes) | 7.1% (1/14) | 10.0% (1/10) | 1.000 |
| Other Antiviral (Yes) | 7.1% (1/14) | 10.0% (1/10) | 1.000 |
| Immunomodulator |  |  |  |
| Systemic Corticosteroids (Yes) | 57.1% (8/14) | 80.0% (8/10) | 0.388 |
| Tocilizumab (Yes) | 14.3% (2/14) | 10.0% (1/10) | 1.000 |
| Sarilumab (Yes) | 7.1% (1/14) | 0.0% (0/10) | 1.000 |
| Other Immunomodulator (Yes) | 21.4% (3/14) | 0.0% (0/10) | 0.239 |
| Other Treatments |  |  |  |
| Vasopressor support (Yes) | 7.1% (1/14) | 10.0% (1/10) | 1.000 |
| Prone Positioning (Yes) | 21.4% (3/14) | 20.0% (2/10) | 1.000 |
| Inhaled Nitric Oxide (Yes) | 0.0% (0/14) | 20.0% (2/10) | 0.163 |
| Blood Transfusion (Yes) | 7.1% (1/14) | 0.0% (0/10) | 1.000 |
| **Follow-up Metrics** | | | |
| Discharge to Follow-up (Days) | 53.6 ± 13.7 (14) | 45.3 ± 15 (10) | 0.135 |
| Mobility Score | 0.1 ± 0.3 (14) | 0.9 ± 0.9 (10) | **0.005** |
| Self-Care Score | 0 ± 0 (14) | 0.2 ± 0.6 (10) | 0.272 |
| Usual Activity Score | 0.1 ± 0.3 (14) | 0.4 ± 0.8 (10) | 0.333 |
| Pain and Discomfort Score | 0.2 ± 0.4 (14) | 1.1 ± 1 (10) | **0.012** |
| Anxiety and Depression Score | 0.1 ± 0.4 (14) | 0.4 ± 0.7 (10) | 0.341 |
| Breathlessness Score | 0.4 ± 0.5 (14) | 1.6 ± 0.8 (10) | **0.001** |
| Difficulty Carrying 10 Pounds | 0.1 ± 0.4 (14) | 0.7 ± 0.9 (10) | 0.113 |
| Difficulty Walking Across Room | 0 ± 0 (14) | 0.2 ± 0.4 (10) | 0.099 |
| Difficulty Climbing 10 Stairs | 0 ± 0 (14) | 0.7 ± 0.8 (10) | **0.004** |
| Difficulty Transfer from Chair to Bed | 0.1 ± 0.3 (14) | 0.3 ± 0.7 (10) | 0.359 |
| Number of Falls in Past Year | 0.1 ± 0.4 (14) | 0.1 ± 0.3 (10) | 0.798 |
| Total Frailty Score | 1.2 ± 1.4 (14) | 6.6 ± 5.8 (10) | **0.002** |
| Poor Health Self-Rating | 15.7 ± 10.2 (14) | 26.6 ± 13.3 (10) | 0.051 |

**Table S3. Patient demographics of patients in each endotype.** For categorical variables, significance was tested using the Chi-squared test with Yates’s correction, or the exact Fisher test if any expected value was <5, and the percentage and fraction of patients fitting the category is displayed. For continuous variables, the Kruskal-Wallis test was used, and the mean ± standard deviation of the variable is displayed, with the number of patients assessed in brackets. Follow-up metric scores are discussed in more detail in **Table S1**. Bolded P-values indicate significant differences (p <0.05) across the three endotypes.

| **Clinical Variables** | **Resolved (12)** | **Suppressive (7)** | **Unresolved (5)** | **P-value** |
| --- | --- | --- | --- | --- |
| Age | 60.5 ± 13.7 (12) | 53.7 ± 13.6 (7) | 56.8 ± 6.1 (5) | 0.535 |
| Sex (Male) | 75.0% (9/12) | 71.4% (5/7) | 60.0% (3/5) | 0.850 |
| Body Mass Index | 33.2 ± 6.9 (10) | 29.3 ± 3.2 (4) | 23.9 ± 2 (5) | **0.010** |
| Admitted to ICU (Yes) | 33.3% (4/12) | 28.6% (2/7) | 20.0% (1/5) | 1.000 |
| Hospitalized Duration (Days) | 18.2 ± 14.8 (12) | 15 ± 17.9 (7) | 10.8 ± 7.6 (5) | 0.381 |
| Post-COVID Symptoms (Yes) | 16.7% (2/12) | 85.7% (6/7) | 40.0% (2/5) | **0.015** |
| Smoker (Yes) | 20.0% (2/10) | 0.0% (0/5) | 25.0% (1/4) | 0.561 |
| **Comorbidities** | | | | |
| Asthma (Yes) | 8.3% (1/12) | 28.6% (2/7) | 0.0% (0/5) | 0.401 |
| COPD (Yes) | 0.0% (0/12) | 14.3% (1/7) | 0.0% (0/5) | 0.500 |
| Chronic Lung Disease (Yes) | 8.3% (1/12) | 14.3% (1/7) | 40.0% (2/5) | 0.285 |
| Atrial Fibrillation/Flutter (Yes) | 16.7% (2/12) | 0.0% (0/7) | 0.0% (0/5) | 0.696 |
| Hypertension (Yes) | 50.0% (6/12) | 14.3% (1/7) | 60.0% (3/5) | 0.263 |
| Heart Failure (Yes) | 8.3% (1/12) | 0.0% (0/7) | 0.0% (0/5) | 1.000 |
| Coronary Artery Disease (Yes) | 16.7% (2/12) | 0.0% (0/7) | 20.0% (1/5) | 0.564 |
| Chronic Cardiac Disease (Yes) | 0.0% (0/12) | 14.3% (1/7) | 40.0% (2/5) | 0.057 |
| Liver disease (Yes) | 0.0% (0/12) | 14.3% (1/7) | 40.0% (2/5) | 0.057 |
| Chronic Hematologic Disease (Yes) | 0.0% (0/12) | 0.0% (0/7) | 20.0% (1/5) | 0.208 |
| Chronic Kidney Disease (Yes) | 25.0% (3/12) | 0.0% (0/7) | 20.0% (1/5) | 0.389 |
| Psychiatric Disease (Yes) | 16.7% (2/12) | 0.0% (0/7) | 20.0% (1/5) | 0.564 |
| Rheumatologic Disease (Yes) | 0.0% (0/12) | 0.0% (0/7) | 0.0% (0/5) | 1.000 |
| Obesity (Yes) | 33.3% (4/12) | 0.0% (0/7) | 0.0% (0/5) | 0.144 |
| Diabetes (Yes) | 50.0% (6/12) | 0.0% (0/7) | 20.0% (1/5) | 0.064 |
| AIDS (Yes) | 8.3% (1/12) | 0.0% (0/7) | 0.0% (0/5) | 1.000 |
| Immunosuppressed (Yes) | 16.7% (2/12) | 0.0% (0/7) | 40.0% (2/5) | 0.206 |
| Cancer (Yes) | 8.3% (1/12) | 0.0% (0/7) | 40.0% (2/5) | 0.168 |
| **Arrival Values** | | | | |
| Temperature (Celsius) | 37.3 ± 0.6 (12) | 37.1 ± 1.5 (7) | 37 ± 0.2 (5) | 0.545 |
| Systolic BP (mmHg) | 128.4 ± 15.4 (12) | 130.6 ± 18.9 (7) | 114.6 ± 15.4 (5) | 0.310 |
| Diastolic BP (mmHg) | 73.2 ± 11.5 (12) | 79.9 ± 6.4 (7) | 73.4 ± 9.4 (5) | 0.298 |
| Respiratory Rate (breaths/min) | 22.2 ± 4 (12) | 25.1 ± 5.5 (7) | 20.4 ± 1.5 (5) | 0.084 |
| Heart Rate (beats/min) | 92.1 ± 12.7 (12) | 98.6 ± 23.6 (7) | 83.6 ± 13.2 (5) | 0.309 |
| O_2_ Saturation at Room Air | 95.9 ± 2.8 (9) | 94.2 ± 2.5 (4) | 94.3 ± 1.5 (3) | 0.461 |
| **Symptoms at Admission** | | | | |
| Asymptomatic (Yes) | 8.3% (1/12) | 0.0% (0/7) | 0.0% (0/5) | 1.000 |
| Confusion (Yes) | 0.0% (0/10) | 14.3% (1/7) | 0.0% (0/4) | 0.524 |
| Diarrhea (Yes) | 30.0% (3/10) | 42.9% (3/7) | 0.0% (0/5) | 0.329 |
| Abdominal Pain (Yes) | 11.1% (1/9) | 0.0% (0/7) | 0.0% (0/5) | 1.000 |
| Chest Pain (Yes) | 40.0% (4/10) | 14.3% (1/7) | 40.0% (2/5) | 0.507 |
| Dyspnea (Yes) | 81.8% (9/11) | 100.0% (7/7) | 60.0% (3/5) | 0.211 |
| Dizziness (Yes) | 10.0% (1/10) | 14.3% (1/7) | 0.0% (0/5) | 1.000 |
| Extremity Numbness (Yes) | 0.0% (0/9) | 28.6% (2/7) | 0.0% (0/5) | 0.148 |
| Fatigue (Yes) | 60.0% (6/10) | 57.1% (4/7) | 0.0% (0/5) | 0.061 |
| Fever (Yes) | 80.0% (8/10) | 57.1% (4/7) | 80.0% (4/5) | 0.592 |
| Hemoptysis (Yes) | 0.0% (0/10) | 28.6% (2/7) | 0.0% (0/5) | 0.134 |
| Loss of Appetite (Yes) | 20.0% (2/10) | 28.6% (2/7) | 0.0% (0/5) | 0.641 |
| Sore Throat (Yes) | 18.2% (2/11) | 14.3% (1/7) | 0.0% (0/5) | 1.000 |
| Headache (Yes) | 27.3% (3/11) | 0.0% (0/7) | 20.0% (1/5) | 0.391 |
| Myalgia (Yes) | 36.4% (4/11) | 0.0% (0/7) | 0.0% (0/5) | 0.106 |
| Nausea/Vomiting (Yes) | 27.3% (3/11) | 42.9% (3/7) | 0.0% (0/5) | 0.321 |
| Loss of Taste/Smell (Yes) | 9.1% (1/11) | 14.3% (1/7) | 20.0% (1/5) | 1.000 |
| Rhinorrhea (Yes) | 9.1% (1/11) | 14.3% (1/7) | 0.0% (0/5) | 1.000 |
| Cough (Yes) | 54.5% (6/11) | 85.7% (6/7) | 80.0% (4/5) | 0.427 |
| Aphasia/Dysphasia (Yes) | 0.0% (0/12) | 0.0% (0/7) | 0.0% (0/5) | 1.000 |
| **Worst Laboratory Values** | | | | |
| Highest %Neutrophil | 81.1 ± 5.7 (12) | 73.6 ± 5.6 (7) | 81.3 ± 12.3 (5) | 0.091 |
| Lowest %Lymphocyte | 5.9 ± 3.0 (12) | 8.6 ± 3.4 (7) | 6.3 ± 3.5 (5) | 0.217 |
| Highest %Monocyte | 9.0 ± 2.4 (12) | 9.6 ± 1.3 (7) | 7.6 ± 2.8 (5) | 0.276 |
| Lowest Platelets (10^3^/µL) | 253 ± 122 (12) | 183 ± 61 (7) | 278 ± 128 (5) | 0.245 |
| Lowest Hemoglobin (g/L) | 105 ± 28 (12) | 129 ± 19 (7) | 121 ± 32 (5) | 0.195 |
| Highest Urea (mg/dL) | 7.8 ± 3.5 (10) | 9.6 ± 4.8 (4) | 12.3 ± 8 (5) | 0.431 |
| Highest Creatinine (µmol/L) | 85.1 ± 42.3 (12) | 63.9 ± 9.8 (7) | 171 ± 192 (5) | **0.034** |
| Highest Sodium (mmol/L) | 142.1 ± 5.5 (12) | 143.3 ± 4.7 (7) | 143.2 ± 5.2 (5) | 0.595 |
| Highest Potassium (mmol/L) | 11.9 ± 21.9 (12) | 4.6 ± 0.5 (7) | 12.9 ± 18.5 (5) | 0.751 |
| Highest AST (units/L) | 87.5 ± 81.8 (8) | 56.5 ± 21.5 (4) | 50.2 ± 29.6 (4) | 0.831 |
| Highest ALT (units/L) | 62.7 ± 54.7 (12) | 63.7 ± 24.8 (6) | 45 ± 32.4 (5) | 0.345 |
| Highest Total Bilirubin (µmol/L) | 15.9 ± 14 (12) | 13.7 ± 6.9 (6) | 10.4 ± 5.3 (5) | 0.697 |
| Highest Glucose (mmol/L) | 15 ± 8.3 (12) | 8.7 ± 3.1 (6) | 14.1 ± 6 (5) | 0.202 |
| Highest Venous Lactate (mmol/L) | 2 ± 1.5 (5) | 2.9 ± 0.4 (2) | 1.4 ± 0.1 (4) | 0.480 |
| Highest D-Dimer (ng/mL) | 3601 ± 4048 (8) | 3120 ± 4741 (5) | 1842 ± 1189 (3) | 0.916 |
| Highest Fibrinogen (g/L) | 8.4 ± 2.4 (7) | 7.9 ± 1.8 (3) | 7.7 ± 1.7 (2) | 0.892 |
| Highest Ferritin (ng/mL) | 509.2 ± 587.8 (5) | 970 ± 463.9 (2) | 286.2 ± 264 (5) | 0.252 |
| Highest C-Reactive Protein (mg/L) | 177.3 ± 85.3 (12) | 115.2 ± 78.5 (7) | 104.5 ± 100 (5) | 0.193 |
| Highest LDH (units/L) | 369.2 ± 97.1 (10) | 301.7 ± 26.6 (3) | 304.2 ± 62.9 (5) | 0.306 |
| Highest Procalcitonin (ng/mL) | 0.2 ± 0.1 (8) | 0.1 ± 0 (5) | 0.9 ± 1.5 (4) | 0.092 |
| Highest Estimated SOFA Score | 3.7 ± 2.8 (12) | 2.9 ± 2.9 (7) | 4.6 ± 3.1 (5) | 0.273 |
| Highest WHO COVID-19 Score | 5.8 ± 1.4 (12) | 5.6 ± 1.6 (7) | 6.0 ± 1.2 (5) | 0.617 |
| **Treatments During Hospitalization** | | | | |
| Antifungal (Yes) | 8.3% (1/12) | 14.3% (1/7) | 0.0% (0/5) | 1.000 |
| Antibiotics | 83.3% (10/12) | 71.4% (5/7) | 80.0% (4/5) | 0.819 |
| Antiviral |  |  |  |  |
| Lopinavir/Ritonavir (Yes) | 33.3% (4/12) | 0.0% (0/7) | 0.0% (0/5) | 0.144 |
| Remdesivir (Yes) | 0.0% (0/12) | 28.6% (2/7) | 0.0% (0/5) | 0.112 |
| Other Antiviral (Yes) | 8.3% (1/12) | 0.0% (0/7) | 20.0% (1/5) | 0.457 |
| Immunomodulator |  |  |  |  |
| Systemic Corticosteroids (Yes) | 41.7% (5/12) | 85.7% (6/7) | 100.0% (5/5) | **0.038** |
| Tocilizumab (Yes) | 0.0% (0/12) | 14.3% (1/7) | 40.0% (2/5) | 0.057 |
| Sarilumab (Yes) | 8.3% (1/12) | 0.0% (0/7) | 0.0% (0/5) | 1.000 |
| Other Immunomodulator (Yes) | 16.7% (2/12) | 0.0% (0/7) | 20.0% (1/5) | 0.564 |
| Other Treatments |  |  |  |  |
| Vasopressor support (Yes) | 8.3% (1/12) | 14.3% (1/7) | 0.0% (0/5) | 1.000 |
| Prone Positioning (Yes) | 25.0% (3/12) | 28.6% (2/7) | 0.0% (0/5) | 0.656 |
| Inhaled Nitric Oxide (Yes) | 0.0% (0/12) | 28.6% (2/7) | 0.0% (0/5) | 0.112 |
| Blood Transfusion (Yes) | 8.3% (1/12) | 0.0% (0/7) | 0.0% (0/5) | 1.000 |
| **Follow-up Metrics** | | | | |
| Discharge to Follow-up (Days) | 54.1 ± 14.2 (12) | 45.7 ± 10 (7) | 46.8 ± 20.4 (5) | 0.395 |
| Mobility Score | 0.3 ± 0.7 (12) | 0.4 ± 0.8 (7) | 0.6 ± 0.9 (5) | 0.805 |
| Self-Care Score | 0.2 ± 0.6 (12) | 0 ± 0 (7) | 0 ± 0 (5) | 0.607 |
| Usual Activity Score | 0.2 ± 0.6 (12) | 0 ± 0 (7) | 0.4 ± 0.9 (5) | 0.494 |
| Pain and Discomfort Score | 0.5 ± 0.9 (12) | 0.9 ± 0.7 (7) | 0.4 ± 0.9 (5) | 0.272 |
| Anxiety and Depression Score | 0.3 ± 0.7 (12) | 0.1 ± 0.4 (7) | 0.2 ± 0.4 (5) | 0.828 |
| Breathlessness Score | 0.7 ± 0.9 (12) | 1.1 ± 0.9 (7) | 1 ± 1 (5) | 0.431 |
| Difficulty Carrying 10 Pounds | 0.3 ± 0.7 (12) | 0.4 ± 0.8 (7) | 0.4 ± 0.9 (5) | 0.967 |
| Difficulty Walking Across Room | 0.1 ± 0.3 (12) | 0 ± 0 (7) | 0.2 ± 0.4 (5) | 0.481 |
| Difficulty Climbing 10 Stairs | 0.2 ± 0.6 (12) | 0.1 ± 0.4 (7) | 0.6 ± 0.9 (5) | 0.475 |
| Difficulty Transfer from Chair to Bed | 0.1 ± 0.3 (12) | 0.1 ± 0.4 (7) | 0.4 ± 0.9 (5) | 0.745 |
| Number of Falls in Past Year | 0.2 ± 0.4 (12) | 0 ± 0 (7) | 0.2 ± 0.4 (5) | 0.500 |
| Total Frailty Score | 3.2 ± 5.2 (12) | 3.3 ± 3.2 (7) | 4.4 ± 5.9 (5) | 0.795 |
| Poor Health Self-Rating | 20.8 ± 11 (12) | 20.1 ± 14.8 (7) | 19 ± 15.6 (5) | 0.927 |

**Table S4. Genes and fold changes specific to each endotype.** Fold changes in bold indicate the top 50 (top 38 for the Suppressive endotype) genes significantly upregulated in that endotype relative to all other endotype (**Figure S11A**), while fold changes in italics indicate non-significant fold changes with absolute fold change <1.5 and/or an adjusted p-value >0.05 from DESeq2. Genes in bold indicate condensed signature genes from LASSO regression (**Figure 4**). RS = Resolved, SP = Suppressive, UR = Unresolved, PG = pseudogene.

| **Gene** | **Fold Change** | | | **Description** |
| --- | --- | --- | --- | --- |
|  | **RS** | **SP** | **UR** |  |
| ENSG00000227242 | **19.9** | *-8.22* | *-3.28* | phosphodiesterase 4D interacting protein PG |
| ENSG00000237846 | **8.59** | *-2.07* | *-8.04* | novel pseudogene |
| ENSG00000241556 | **7.88** | *-1.92* | NA | ribosomal protein L29 (RPL29) PG |
| ENSG00000274422 | **7.40** | *-7.33* | *-3.58* | novel transcript |
| ERFE | **6.21** | *-5.87* | *-4.76* | erythroferrone |
| RPS3AP26 | **5.30** | *-3.43* | *-5.28* | RPS3A pseudogene 26 |
| S100B | **4.56** | *-1.35* | -6.00 | S100 calcium binding protein B |
| PFDN4 | **4.37** | *-4.48* | *-2.49* | prefoldin subunit 4 |
| TUBB2A | **4.23** | *-1.99* | *-4.62* | tubulin beta 2A class IIa |
| TIPARP-AS1 | **4.16** | *-3.13* | *-2.88* | TIPARP antisense RNA 1 |
| RPL36AP37 | **4.01** | *-3.67* | *-2.89* | ribosomal protein L36a PG 37 |
| LOC101927039 | **3.96** | -4.50 | *-2.23* | uncharacterized LOC101927039 |
| CENPK | **3.92** | *-3.27* | -2.91 | centromere protein K |
| RPL34 | **3.91** | *-2.99* | -3.68 | ribosomal protein L34 |
| RPL26 | **3.85** | *-3.01* | -3.36 | ribosomal protein L26 |
| CETN3 | **3.77** | *-3.43* | -2.92 | centrin 3 |
| UQCRB | **3.40** | -3.38 | *-2.32* | ubiquinol-cytochrome c reductase binding protein |
| RPL31 | **3.37** | *-2.57* | -3.09 | ribosomal protein L31 |
| MIR155HG | **3.36** | *-2.47* | -3.60 | MIR155 host gene |
| MICU3 | **3.35** | *-2.60* | -4.29 | mitochondrial calcium uptake family member 3 |
| **LRRCC1** | **3.31** | -2.92 | *-2.19* | leucine rich repeat and coiled-coil centrosomal protein 1 |
| FSBP | **3.30** | *-2.54* | *-3.00* | fibrinogen silencer binding protein |
| RPS7P1 | **3.26** | *-2.50* | -2.86 | ribosomal protein S7 pseudogene 1 |
| ENSG00000258732 | **3.25** | *-1.99* | -3.03 | arginine-glutamic acid dipeptide (RE) repeats PG |
| L1TD1 | **3.21** | *-1.26* | -8.41 | LINE1 type transposase domain containing 1 |
| **KLRC1** | **3.21** | *-1.88* | -2.88 | killer cell lectin like receptor C1 |
| ZNF711 | **3.20** | *-2.24* | -3.19 | zinc finger protein 711 |
| RPS24 | **3.17** | *-2.56* | -2.53 | ribosomal protein S24 |
| RPS3AP21 | **3.13** | *-2.23* | -3.77 | RPS3A pseudogene 21 |
| COMMD6 | **3.10** | *-2.41* | -2.69 | COMM domain containing 6 |
| LERFS | **3.06** | *-3.27* | *-1.63* | lncRNA negative regulator of fibroblast-like synoviocyte migration, SYNCRIP interacting |
| **RPS3AP6** | **3.01** | *-2.37* | -2.41 | RPS3A PG 6 |
| TRDV2 | **3.00** | *-2.74* | *-1.99* | T cell receptor delta variable 2 |
| **NDUFA5** | **2.99** | *-2.54* | -2.35 | NADH:ubiquinone oxidoreductase subunit A5 |
| ASAH2B | **2.98** | *-2.18* | -2.49 | N-acylsphingosine amidohydrolase 2B |
| SH3D19 | **2.95** | *-2.19* | -2.81 | SH3 domain containing 19 |
| **GRPEL2-AS1** | **2.93** | *-1.70* | -2.76 | GRPEL2 antisense RNA 1 |
| LOC107986115 | **2.91** | *-1.98* | -2.67 | uncharacterized LOC107986115 |
| SLC4A4 | **2.90** | *-2.23* | *-2.20* | solute carrier family 4 member 4 |
| ICOSLG | **2.89** | *-1.23* | *-2.80* | inducible T cell costimulator ligand |
| **HECTD2** | **2.89** | *-2.22* | *-2.44* | HECT domain E3 ubiquitin protein ligase 2 |
| MAP9 | **2.89** | *-1.85* | -3.19 | microtubule associated protein 9 |
| IFNG | **2.88** | *-2.45* | *-2.33* | interferon gamma |
| RPL7P9 | **2.87** | *-2.38* | -2.37 | ribosomal protein L7 PG 9 |
| **LINC02446** | **2.82** | *-2.82* | *-1.88* | long intergenic non-protein coding RNA 2446 |
| ZNF204P | **2.82** | *-1.90* | -3.15 | zinc finger protein 204 PG |
| RPS3A | **2.81** | *-2.10* | -2.68 | ribosomal protein S3A |
| ENSG00000287097 | **2.80** | *-2.53* | *-1.61* | NA |
| CD69 | **2.80** | *-2.09* | -2.64 | CD69 molecule |
| SCOC | **2.78** | *-2.28* | -2.40 | short coiled-coil protein |
| IGLV10-54 | *-2.84* | **16.3** | *-4.29* | immunoglobulin lambda variable 10-54 |
| IGHV3-73 | *-2.36* | **5.51** | *-1.78* | immunoglobulin heavy variable 3-73 |
| IGHV1-2 | *-2.29* | **4.80** | *-1.71* | immunoglobulin heavy variable 1-2 |
| IGLV5-45 | *-2.20* | **4.27** | *-1.90* | immunoglobulin lambda variable 5-45 |
| IGHG2 | *-2.18* | **4.15** | *-2.28* | immunoglobulin heavy constant gamma 2 |
| **IGKV1-27** | *-1.96* | **3.41** | *-1.42* | immunoglobulin kappa variable 1-27 |
| **DBNDD1** | *-1.10* | **3.04** | -4.32 | dysbindin domain containing 1 |
| **CEACAM19** | *-1.76* | **2.65** | *-1.52* | CEA cell adhesion molecule 19 |
| PPFIA4 | -1.82 | **2.48** | *-1.38* | PTPRF interacting protein alpha 4 |
| NLGN2 | *-1.34* | **2.41** | -2.11 | neuroligin 2 |
| ERFL | *-1.45* | **2.37** | -1.91 | ETS repressor factor like |
| LINC01238 | *-1.20* | **2.27** | -2.30 | long intergenic non-protein coding RNA 1238 |
| ENSG00000260496 | *-1.42* | **2.12** | *-1.65* | novel transcript |
| HSPG2 | *-1.27* | **2.10** | -1.92 | heparan sulfate proteoglycan 2 |
| TNNT3 | *-1.57* | **2.04** | *-1.33* | troponin T3, fast skeletal type |
| S100A13 | *-1.48* | **1.88** | *-1.26* | S100 calcium binding protein A13 |
| ALS2CL | *-1.04* | **1.86** | -2.17 | ALS2 C-terminal like |
| **CACNA1I** | *-1.15* | **1.86** | -1.86 | calcium voltage-gated channel subunit alpha1 I |
| ANO9 | *-1.13* | **1.85** | -1.88 | anoctamin 9 |
| LTBP3 | *-1.30* | **1.85** | *-1.48* | latent transforming growth factor β binding protein 3 |
| REEP6 | *-1.20* | **1.84** | -1.68 | receptor accessory protein 6 |
| GSTM2 | *-1.32* | **1.80** | *-1.50* | glutathione S-transferase mu 2 |
| **ADAMTSL5** | *-1.27* | **1.79** | -1.52 | ADAMTS like 5 |
| ATG9B | *-1.13* | **1.78** | -1.77 | autophagy related 9B |
| CDC42BPG | *-1.13* | **1.76** | -1.71 | CDC42 binding protein kinase gamma |
| ASIC3 | *-1.35* | **1.74** | *-1.31* | acid sensing ion channel subunit 3 |
| ENGASE | *-1.29* | **1.71** | *-1.33* | endo-beta-N-acetylglucosaminidase |
| SORBS3 | *-1.17* | **1.71** | -1.59 | sorbin and SH3 domain containing 3 |
| ENSG00000254230 | *-1.10* | **1.69** | -1.73 | novel transcript |
| TPCN1 | *-1.25* | **1.58** | *-1.26* | two pore segment channel 1 |
| DGKA | *-1.19* | **1.58** | *-1.36* | diacylglycerol kinase alpha |
| ENSG00000245261 | -1.37 | **1.56** | *-1.09* | novel transcript |
| MRPL38 | *-1.13* | **1.54** | -1.43 | mitochondrial ribosomal protein L38 |
| **AMN** | *-1.24* | **1.54** | *-1.24* | amnion associated transmembrane protein |
| SGSM3 | *-1.31* | **1.54** | *-1.15* | small G protein signaling modulator 3 |
| ZNF512B | *-1.05* | **1.54** | -1.59 | zinc finger protein 512B |
| INF2 | *-1.25* | **1.51** | *-1.18* | inverted formin 2 |
| MORC2-AS1 | *-1.21* | **1.51** | *-1.24* | MORC2 antisense RNA 1 |
| **PCSK9** | -186 | *-4.14* | **949** | proprotein convertase subtilisin/kexin type 9 |
| LOC107987373 | -212 | *-2.92* | **47.4** | 39S ribosomal protein L23, mitochondrial |
| ADAMTS2 | *-4.21* | *-2.47* | **39.0** | ADAM metallopeptidase thrombospondin type 1 motif 2 |
| DAAM2-AS1 | -6.72 | *-1.74* | **18.6** | DAAM2 antisense RNA 1 |
| ENSG00000272396 | -7.66 | *-5.52* | **18.4** | novel transcript, antisense to CD177 |
| CD177 | -5.08 | *-7.76* | **16.2** | CD177 molecule |
| CLRN1-AS1 | -8.33 | *-2.24* | **15.4** | CLRN1 antisense RNA 1 |
| DAAM2 | -6.99 | *-1.03* | **14.2** | dishevelled associated activator of morphogenesis 2 |
| ENSG00000231412 | -5.88 | *-3.83* | **13.9** | novel transcript |
| LIPM | -5.71 | *-4.50* | **11.6** | lipase family member M |
| ANKRD22 | *-3.42* | *-4.17* | **10.3** | ankyrin repeat domain 22 |
| LINC01093 | *-3.32* | *-5.88* | **10.1** | long intergenic non-protein coding RNA 1093 |
| CCNA1 | *-3.25* | *-3.08* | **10.0** | cyclin A1 |
| ENSG00000253772 | -4.07 | *-2.25* | **9.83** | acid phosphatase 1, soluble (ACP1) PG |
| NAV3 | *-4.21* | *-1.92* | **9.80** | neuron navigator 3 |
| H2BP2 | *-3.38* | *-3.41* | **9.32** | H2B histone PG 2 |
| **CD300LD** | -5.82 | *-1.61* | **9.31** | CD300 molecule like family member d |
| ENSG00000254859 | *-3.74* | *-2.93* | **9.07** | novel transcript |
| ENSG00000254695 | -4.57 | *-3.56* | **8.99** | novel transcript |
| ENSG00000243273 | -3.64 | *-2.58* | **8.88** | novel transcript |
| OLAH | *-2.16* | *-3.23* | **8.83** | oleoyl-ACP hydrolase |
| FLT1P1 | -5.56 | *-1.70* | **8.79** | FLT1 PG 1 |
| ENSG00000270972 | -3.54 | *-3.11* | **8.38** | novel transcript |
| GPR84 | -4.08 | *-2.57* | **8.37** | G protein-coupled receptor 84 |
| **SOCS3-DT** | -3.15 | *-2.97* | **8.31** | SOCS3 divergent transcript |
| MIR3945HG | -4.69 | *-2.57* | **8.15** | MIR3945 host gene |
| **CYP19A1** | *-3.25* | *-2.61* | **8.14** | cytochrome P450 family 19 subfamily A member 1 |
| **C4BPA** | -4.39 | *-2.47* | **8.03** | complement component 4 binding protein alpha |
| DPRXP2 | -4.81 | *-2.18* | **7.70** | divergent-paired related homeobox pseudogene 2 |
| DEFA8P | *-2.90* | *-2.39* | **7.61** | defensin alpha 8, pseudogene |
| GALNT14 | -4.40 | *-2.30* | **7.61** | polypeptide N-acetylgalactosaminyltransferase 14 |
| ETV7 | -4.05 | *-1.97* | **7.58** | ETS variant transcription factor 7 |
| ENSG00000286256 | -4.32 | *-2.55* | **7.58** | NA |
| METTL7B | -3.40 | *-2.30* | **7.50** | methyltransferase like 7B |
| SERPING1 | -4.56 | *-2.03* | **7.48** | serpin family G member 1 |
| ENSG00000255328 | -2.76 | *-3.37* | **7.43** | novel transcript |
| LINC02751 | -5.69 | *-1.47* | **7.40** | long intergenic non-protein coding RNA 2751 |
| MOCS1 | -4.87 | *1.63* | **7.22** | molybdenum cofactor synthesis 1 |
| HDC | -3.91 | *-2.59* | **7.18** | histidine decarboxylase |
| NSG2 | -3.58 | *-2.06* | **6.98** | neuronal vesicle trafficking associated 2 |
| FCGR1CP | *-2.70* | *-2.87* | **6.96** | Fc fragment of IgG receptor Ic, pseudogene |
| SLC26A8 | -3.30 | *-2.56* | **6.94** | solute carrier family 26 member 8 |
| CBS | -3.89 | *1.24* | **6.89** | cystathionine beta-synthase |
| FCGR1A | *-2.65* | *-2.96* | **6.81** | Fc fragment of IgG receptor Ia |
| ANOS1 | *-3.54* | *-1.16* | **6.81** | anosmin 1 |
| PI3 | -3.11 | *-2.29* | **6.75** | peptidase inhibitor 3 |
| GK-IT1 | -5.18 | *-1.33* | **6.72** | GK intronic transcript 1 |
| KREMEN1 | *-2.76* | *-3.59* | **6.62** | kringle containing transmembrane protein 1 |
| **ENSG00000251139** | -3.97 | *-1.49* | **6.59** | novel transcript, antisense to ACSL1 |
| ENSG00000254789 | *-3.07* | *-2.70* | **6.57** | novel transcript |
